# Comprehending radicals, diradicals and their bondings in aggregates of imide-fused polycyclic aromatic hydrocarbons†

**DOI:** 10.1039/d2sc02906e

**Published:** 2022-08-06

**Authors:** Jiani Liu, Jiarui Zeng, Duokai Zhao, Yao Yao, Dehua Hu, Yuguang Ma

**Affiliations:** Department of Physics, South China University of Technology Guangzhou 510640 China yaoyao2016@scut.edu.cn; State Key Laboratory of Luminescent Materials and Devices, South China University of Technology Guangzhou 510640 China

## Abstract

Quantum effects such as ferromagnetism were regarded as rare in organic materials. When reduced to radical states, imide-fused polycyclic aromatic hydrocarbons (IPAHs) have shown room-temperature ferromagnetism in our recent work, to be a potential candidate as ferromagnetic semiconductor. Here, we use variational Davydov ansatz parametrized by density functional theory to investigate the structural and optical properties of IPAHs and their radicals at both molecule and aggregate levels. Our calculation reveals that hydrogen mainly gives rise to radicals and proves the formation of a mid-gap polaronic state, which is further evidenced by UV-vis absorption spectra simulations, in good agreement with experiments. The significant change of dispersion between the π–π stacking structure and planar structure implies the formation of radical–radical bonding (pancake bonding), which is revealed by simulations of NIR absorption signals and serves as the physical basis of long-range ferromagnetic orders. Absorption spectra of perylene diimide (PDI), terrylene diimide (TDI) and their radicals are also predicted.

## Abstract 2

The structures and optical properties of imide-fused polycyclic aromatic hydrocarbon radicals, which exhibit room-temperature ferromagnetism as discovered recently and the strong pancake bondings in NDI radical aggregates are investigated.

## Graphical abstract

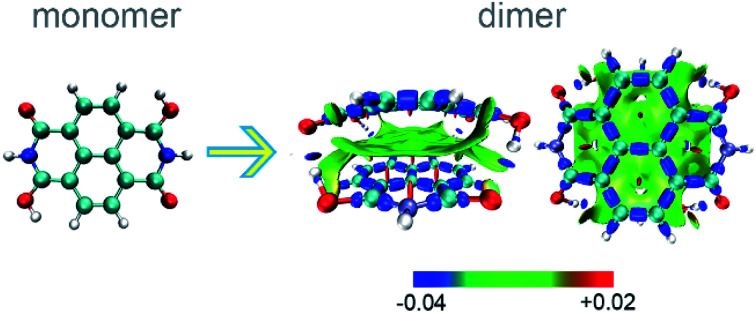

## Introduction

By introducing electron-withdrawing groups such as halogens, cyano groups and carbonyl groups, polycyclic aromatic hydrocarbons can be modified to function as electron acceptors. The great progress of organic solar cells in recent years was possible due to the blooming new kinds of acceptors.^1,2^ Imide-fused hydrocarbons serve as common candidates for acceptors due to the two carbonyl groups and the perfect planarity of molecules, and the strong hydrogen bonds make them aggregate easily leading to a pretty rigid structure. The advantage of these materials is the good π–π stacking features, but the disadvantage is the poor solubility and miscibility. A common strategy to overcome this disadvantage is to add alkyl chains, which will however weaken the excellent structure and properties. For example, Margulies *et al.*^3^ considered a series of covalently linked TDI dimers in which triptycene spacers held the two TDI molecules in a series of π-stacked geometries, and investigated the role of charge transfer in singlet fission. Wang *et al.*^4^ synthesized a series of PDI-based small molecular acceptors in which 9H-fluorene was used as the central core and two PDI units or PDI dimer units as the terminal groups. In the current stage, a new route to compromise the contradiction in imide-fused hydrocarbons would always be helpful in wider applications of these materials. A possible idea is to utilize radicals.^5–7^*Via* reduction, the oxygen on the carbonyl group may extract an electron and a carbon radical emerges. Assisted by the conjugated π orbitals on hydrocarbons, the radical could be delocalized and stabilized. In terms of the subsequent polarization of the molecule, the solubility could be largely increased making the solution processes feasible. More importantly, this method will not weaken the rigidity of IPAHs so that it is an optimized strategy to compromise the solubility and appealing quantum effects.^5–7^

Optical and magnetic response turns out to be the essential basis of radicals in all applications of organic electronics and spintronics.^8^ Research on the spin degree of freedom in organic materials has a long history. The majority of them such as singlet fission refer to organic crystals, which are usually annealed in order to be sufficiently ordered.^9^ Thin films directly fabricated from the solution phase are normally amorphous and the carriers in organic molecules with a closed shell are then intuitively regarded to be polarons which are localized electrons self-trapped by molecular vibrations.^10,11^ In inorganic systems, on the other hand, the band gap is the sole quantity used to distinguish metals and semiconductors. The organic molecular aggregates normally do not have a well-defined energy band, so that we should take the strong vibronic couplings into considerations. A dynamic perspective, namely a framework combining both coherent and incoherent dynamics then reasonably works.^12^ The thermal fluctuation weakens the former one and prefers the latter, such that the stochastic motion of charge and spin dominates the microscopic dynamics in organic materials, manifesting a novel magnetic response.^13^ The temperature effect of magnetism in a fabricated film purely through solution-processed treatment then significantly enables us to comprehend the mechanism.^14^

Since the radicals normally delocalize to certain π-conjugated molecules and even form radical bonds with other radicals, they share similar features with the large polarons, which stem from strong vibronic couplings and have fermionic statistical properties with spin half.^15^ As long as the vibronic couplings are strong enough to suppress the Coulomb repulsion among electrons, there emerge another elementary excitation, which can be named bipolarons or diradicals.^16^ Since two noninteracting radicals go together into a single molecular potential trap to form a diradical, the fermions are converted to bosons so that the spin of diradicals normally vanishes.^17,18^ This implies that the magnetic responses of two radicals and diradicals would be significantly different. In addition, due to the bosonic features of diradicals, Pauli's exclusion principle does not work and their statistical distribution will be essentially different from the fermionic distribution, such that the theoretical framework of the energy band is not able to essentially describe the charge and spin properties of diradicals. So far, how the diradicals influence the features of materials deserves much more comprehensive research.

In this work, density functional theory (DFT) is used to calculate the electronic structures of IPAHs and their radicals. Variational Davydov ansatz, time-dependent density functional theory (TDDFT) and TDDFT with Franck–Condon analysis (FC-TDDFT) are used to calculate the excitation dynamics and simulate the UV-vis absorption spectrum.^19–21^ We find that hydrogen mainly results in the radicals and the absorption spectrum of the NDI diradical formed by introducing double H is in good agreement with the experiment. Experimentally, IPAH radicals combine into aggregates through powerful interaction and produce room temperature ferromagnetism. Through single point calculation with counterpoise correction and symmetry-adapted perturbation theory (SAPT) for NDI radical dimers and tetramer, the major part of radical–radical pancake bonding for interaction in aggregates is revealed. NIR spectra of the NDI anion diradical dimer by TDDFT and Davydov ansatz qualitatively agree with experimental results and further confirm the characteristics of pancake bonding. Davydov ansatz is used to further predict the absorption spectra of PDI, TDI and their radicals whose structures are similar to NDI but have more atoms.

## Results and discussion

### Charge and spin properties of NDI and their radicals

**Fig. 1:**
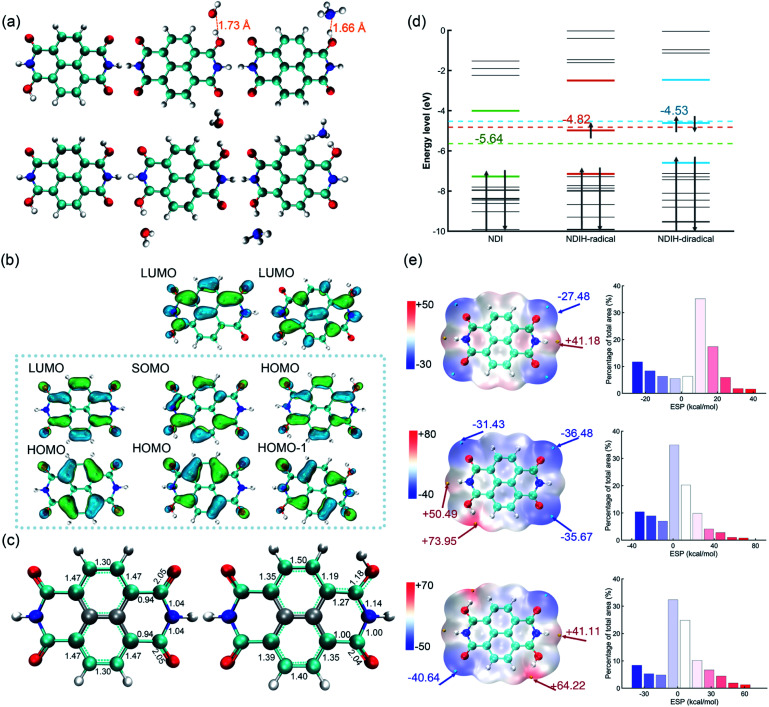
(a) Optimized structure of the NDIH-radical, NDIH-diradical, NDIH_3_O-radical, NDIH_3_O-diradical, NDINH_4_-radical and NDINH_4_-diradical at the HSE06/6-311G** level. Red, blue, cyan and white spheres represent O, N, C and H, respectively. (b) Molecular orbital images of NDI, NDIH-radical and NDIH-diradical from left to right (isovalue = 0.045 atomic.u.). Green and blue regions represent isovalue positive and negative areas, respectively. (c) Optimized structure and Mayer bond order of NDI and NDIH-radical. (d) Energy level diagram of NDI, NDIH-radical and NDIH-diradical from −10 eV to 0 eV. Dotted lines and numbers indicate the central positions and energies of NDI HOMO–LUMO, NDI-radical HOMO–LUMO and NDI-diradical HOMO-1-LUMO, respectively. (e) ESP and ESP distribution of NDI, NDIH-radical and NDIH-diradical. The cyan and yellow points represent minimum and maximum ESP values, respectively. Isosurfaces are *ρ* = 0.001 atomic.u.

In the experiment, NDI is dissolved and ionized in hydrazine hydrate solution. After dropping onto a film, room-temperature ferromagnetism is observed.^14^ It is noteworthy that ferromagnetism is caused by radicals and the candidate groups existing in hydrazine hydrate solution that can provide electrons might be H, H_3_O and NH_4_. Moreover, because the carbonyl groups at both ends of NDI have the ability of strongly attracting electrons, radicals might be generated at these sites. To this end, we consider introducing single or double H, H_3_O and NH_4_ groups on these positions and name them the NDIH-radical, NDIH-diradical, NDIH_3_O-radical, NDIH_3_O-diradical, NDINH_4_-radical and NDINH_4_-diradical, respectively (Fig. 1a). As we can see, for optimized structures, the introduced H atoms tend to bond with NDI. The hydrogen atoms on H_3_O or NH_4_ choose to hybridize with the oxygen atom on NDI carbonyl, while the remaining H_2_O or NH_3_ molecules separate away from the newly formed molecule. For example, in the NDIH_3_O-radical, the distance between the O in the separated H_2_O and the H combined with NDI is 1.73 Å, which is greater than the O–H bond length of 0.98 Å. In the NDINH_4_-radical, the distance between the N in the separated NH_3_ and the H combined with NDI is 1.66 Å and the N–H bond length is 1.01 Å, exhibiting a similar feature to the NDIH_3_O-radical. For H_2_O and NH_3_ which are far away from NDI, their direction is random as shown in the figure. We can then conclude here that it is the hydrogen atoms that introduce radicals into NDI.

For describing diradical characteristics, it is common to construct spin polarized singlet states. Rather, the quantification of diradical characteristics strongly depends on theoretical levels. In our calculation, although the spin polarized singlet state of the NDIH-diradical can be described at the UHF/6-311G** level (Fig. S2†),^22^ at the DFT level (HSE06/6-311G** in this calculation), even if the initial state with symmetry breaking is constructed, the optimized wave function converges to the singlet state with vanishing spin density all the way, and the diradical characteristics cannot be well described in this theoretical level. We think this may be caused by the nontrivial topology of electronic structures in terms of the production of diradicals. Hence, we draw the frontier molecular orbital images of NDI, NDIH-radical and NDIH-diradical for comparison (Fig. 1b). For NDI HOMO and LUMO, as indicated by the dashed box, the charge is concentrated on the outer ring of naphthalene to form a quasi-one-dimensional ring with alternative single and double bonds. This is more like a polymer chain with few repeating units. With the introduction of H, the charge in the NDI HOMO on naphthalene moves towards the carbonyl group, the charge on O connected to H decreases, and charge accumulates on N on the side of radical generation, as shown in the NDIH-radical HOMO and NDIH-diradical HOMO-1. In the NDIH-radical SOMO and NDIH-diradical HOMO, part of the charge in the NDI LUMO on the end group C of NDI LUMO is transferred to the adjacent N. Considering the similar situations in conducting polymers, these charge transfer mechanisms give rise to self-doping and thus the formation of polarons and bipolarons in the chain.

To examine this scenario, the change of charge density induced by the radical is described by the change of optimized structural bond lengths and Mayer bond orders, taking NDI and NDIH-radical as examples (Fig. 1c). The number between atoms in the figure represents the Mayer bond order. The bond marked by the cyan dotted line indicates that the bond length lies between single and double bond lengths. The bonds and atoms in the center of naphthalene are represented by grey color as the changes of bond orders on these sites are weak. The bond orders of C–O, C–C and C–N bonds change significantly from 2.05, 0.94 and 1.04 of NDI to 1.18, 1.27 and 1.14 of the NDIH-radical, and from double bond, single bond and single bond to the bond between single and double bonds. The charge distributed in the naphthalene outer ring of NDI is no longer symmetric in the NDIH-radical, which implies that the Mayer bond orders above and below the naphthalene outer ring of NDIH-radical are no longer equal. This result clearly indicates that the self-doping mechanism leads to spatial localization and breaks the original translational invariance of electronic orbitals in neutral molecules. A polaron or bipolaron is then generated in the radical or diradical phase. We believe that this scenario can be applied to interpret the exotic room-temperature ferromagnetism, and other strongly correlated effects such as superconductivity may also be possible in these pronounced electronic structures.

Energy level diagrams can also be made to evidence the above mechanism (Fig. 1d). For the NDIH-radical, SOMO, −4.98 eV, is very close to the mid-gap of HOMO and LUMO, which is −4.82 eV. The mid-gap energy level is the unique feature of soliton-like polarons in conducting polymers.^15^ For the NDIH-diradical, the mid-gap of HOMO-1 and LUMO is −4.53 eV, near the HOMO, −4.61 eV, which is the energy level occupied by two polarons.

For determining the spin multiplicity of the system at the HSE06/6-311G** level, we compare the energies of the NDIH-diradical, NDIH_3_O-diradical, NDINH_4_-diradical singlet and triplet states (Table S18†). It is observed that all singlet state energies are lower than triplet state energies. α electrons are all concentrated on NDI introduced hydrogen atoms (Fig. S3†), which further proves that radicals are introduced by hydrogen. Since the singlet state has lower energy, ferromagnetism should be recognized as a dynamically stable phase as discussed in the Introduction section.

Electrostatic potential (ESP) is a physically observable quantity and commonly used to describe the charge distribution.^23–25^ Multiwfn, which has significant advantages in efficiently calculating ESP,^26–28^ is used for analyzing ESP in our systems (Fig. 1e). The isosurface is van der Waals (vdW) surface with *ρ* = 0.001 atomic.u. The ESP of NDI is symmetric. The electronegativity of O on the four end groups is strong, which makes the ESP near these sites the most negative. The cyan points are the minimum values of NDI ESP, −27.48 kcal mol^−1^, while the yellow points are the maximum values, 41.18 kcal mol^−1^. After introducing additional hydrogen, the ESP changes greatly. For the NDIH-radical, the ESP maximum point appears at the position of H, and the minimum point is away from the radical. The NDIH-diradical ESP exhibits central symmetry as expected. Molecular surface ESP distribution images are also given.^29,30^ The vertical axis represents the percentage of ESP area in the region on the *X*-axis in the total ESP area. The introduced H makes ESP distribution wider, and the negative ESP region increases. The change of ESP can further explain the presented structures of NDI aggregates.

**Fig. 2:**
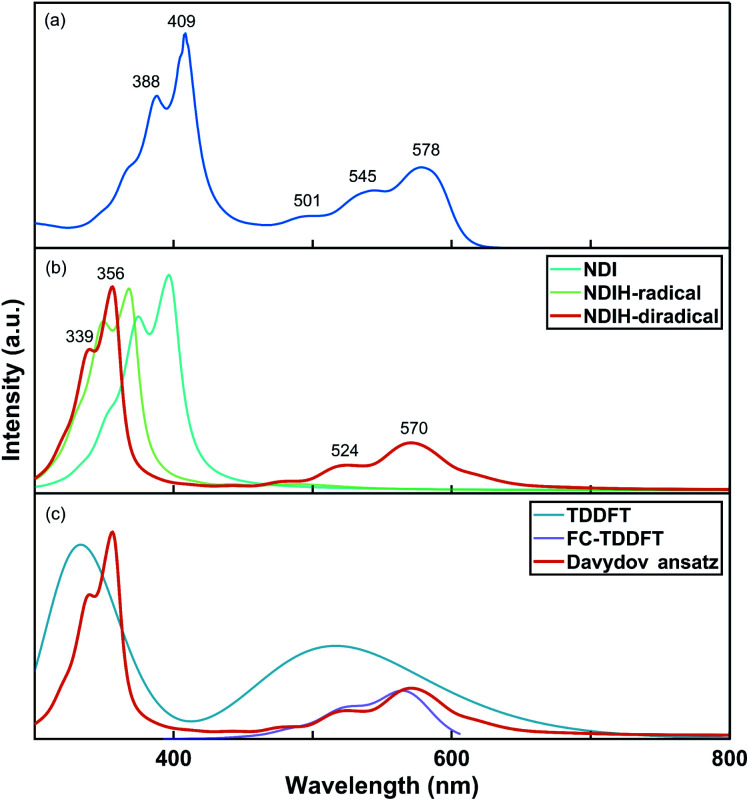
(a) Experimental UV-vis absorption spectrum of the NDI ionized derivative. (b) Simulated UV-vis absorption spectra of NDI, NDIH-radical and NDIH-diradical by Davydov ansatz. (c) Simulated UV-vis absorption spectra of the NDIH-diradical by TDDFT, FC-TDDFT and Davydov ansatz, respectively.

The UV-vis absorption spectrum of the NDI ionized derivative is measured experimentally (Fig. 2a). Because the aggregation of NDI in the solution phase is very easy, the solution is diluted to such an extent that there are no obvious signals of aggregates in the UV-vis absorption measurement. A main peak can be seen at 409 nm with a side peak at 388 nm. The other main peak appears at 578 nm with two side peaks at 545 and 501 nm. However, the structure of NDI ionized derivative could not be directly detected in the experiment. So, we attempted to confirm the structure by comparing the spectrum of the experiment with that of NDI radicals. Davydov ansatz is used to calculate the spectra of NDI, NDIH-radical and NDIH-diradical (Fig. 2b). For NDI, 0–0 transition is at 397 nm and 0–1 transition is at 375 nm. NDIH-radical has a strong 0–0 transition at 367 nm with a side peak at 349 nm and very weak absorption peaks at 441 and 490 nm. We suggest that the huge intensity peak difference is due to oscillator strength which cannot be described well when the radical exists and the problem will be discussed in the future. NDIH-diradical has two main peaks at 356 nm and 570 nm, and the corresponding 0–1 transition at 339 nm and 524 nm, which agree with the experiment. The distance between the two main peaks is greater than that of the experiment because the band gaps calculated based on the HSE06/6-311G** level are overestimated in this system. More details can be found in the Functional Test section of the ESI.† Besides, our calculation is simulated in vacuum, which also affects the spectrum. The absorption spectra of the NDIH_3_O-diradical and NDINH_4_-diradical are also calculated by Davydov ansatz (Fig. S4†). It is observed that the spectra of the NDIH-diradical and NDINH_4_-diradical are both in good agreement with the experiment. For the NDIH_3_O-diradical, even changing the broadening of the side peaks cannot be effectively described. The distance between the two main peaks of the NDINH_4_-diradical is 228 nm, slightly larger than the 214 nm of the NDIH-diradical. It may be caused by the free NH_3_ group. Through the observation of the absorption spectrum, we can conclude here that the NDIH-diradical is the structure of the NDI ionized derivative and we call it NDIHs in the following calculation.

For further testing the accuracy of the Davydov ansatz method, we compare the absorption spectrum of NDIHs obtained by Davydov ansatz with those by TDDFT and FC-TDDFT (Fig. 2c). As we can see, TDDFT cannot effectively describe the side peaks caused by vibrations. FC-TDDFT can only calculate one absorption peak for one run. While describing the vibration resolved spectrum at short wavelength, the result is prompted unreliable and the absorption peak cannot be accurately described. By using the Davydov ansatz method, two main absorption peaks can be obtained in the meantime, and the peak caused by radicals is close to that by FC-TDDFT. We then state that Davydov ansatz has more advantages in calculating the absorption spectrum of this molecule.

### Pancake bonding in NDI radical aggregates

Pancake bonding originates from π–π stacking between monomer radicals and leads to more attractive interaction and shorter intermolecular contact distances than those of vdW interaction.^31–33^ The combination between IPAH aggregates is strong, which may be due to the pancake bonding. In addition, the electronic and vibronic interaction in different sizes of aggregates change significantly.^34^ They essentially affect the self-trapping polaronic states and thus the observed ferromagnetism in the experiment. To further explore the pancake bonding of IPAHs after introducing radicals, we investigate the dimers and tetramer formed by NDIHs.

**Fig. 3:**
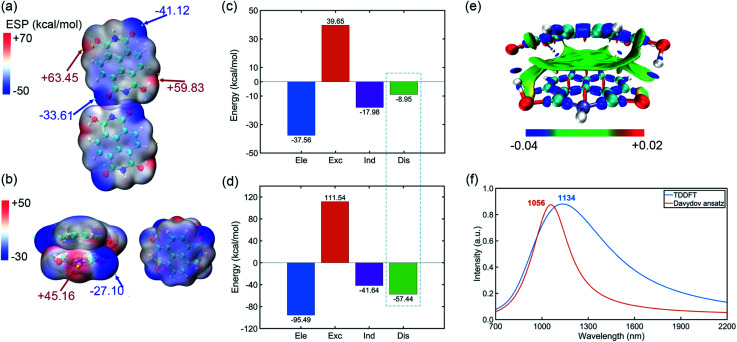
(a) ESP of the NDIH horizontal dimer. (b) ESP of the NDIH vertical dimer. ESP isosurfaces are both *ρ* = 0.001 atomic.u. (c) Components of interaction energy in the NDIH horizontal dimer by SAPT calculation. Ele, Exc, Ind and Dis represent electrostatic, exchange, induction and dispersion energy, respectively. (d) IRI of the NDIH vertical dimer. Isovalue = 1.0 atomic.u. (e) Components of interaction energy in the NDIH vertical dimer by SAPT calculation. (f) Simulated NIR absorption spectra of the NDIH anion vertical dimer by TDDFT and Davydov ansatz.

The optimized structures of horizontal and vertical NDIH dimers are shown in the ESI (Fig. S5†). It is observed that energies of NDIH vertical dimers vary greatly with the monomer directions (Fig. S5b†). We mainly focus on the structure with the lowest energy and take the structure with an initial slip of 4.0 Å in the *Y* direction as the NDIH vertical dimer in the following. The ESP shows the charge distribution of NDIH horizontal and vertical dimers (Fig. 3a, b). For the NDIH horizontal dimer, two monomers are close to each other in the direction of hydrogen bond formation, as shown in the overlapping part of red and blue in the ESP. For the NDIH vertical dimer, the ESP region with the largest change overlaps and the strong attraction makes monomers bend decreasing the geometric center distance to 2.77 Å. Compared with the horizontal dimer, there are more overlapping areas of positive and negative ESP in the vertical dimer. Moreover, the bonding and antibonding combinations of four molecular orbitals, LUMO, LUMO+1, LUMO+2 and LUMO+3 show the overlaps of electron clouds and represent the pancake bond order = 2 in the vertical dimer (Fig. S7†).

Interactions between these monomers turn out to be a significant key for us to understand radical–radical bondings as well as the long-range ordering of ferromagnetism. B3LYP-D3BJ/6-311+G** combined with Counterpoise correction is used for interaction energy calculation.^35–40^ It can be found that the interaction energy of the NDIH vertical dimer is −73.70 kcal mol^−1^, which is much lower than the −22.73 kcal mol^−1^ of the NDIH horizontal dimer (Table S20†). SAPT is often used to analyze the component of interaction energy for the dimer.^41–44^ PSI4 is used for SAPT calculation at the level of sSAPT0/Jun-cc-PVDZ and the input files are generated with the help of the Multiwfn program.^26,45,46^ The interaction energies obtained by SAPT are slightly lower than those obtained by single point calculation (Table S20†). For the NDIH horizontal dimer, the interaction energy obtained by SAPT is −24.84 kcal mol^−1^ and for the NDIH vertical dimer, it is −83.03 kcal mol^−1^. The interactions of the NDIH vertical dimer obtained by both methods are much stronger than those of the NDIH horizontal dimer, which further proves that dimers can easily form vertical stacking and thus the pancake bonding. In addition, the electrostatic term contributes the most to the attraction energy, which is −37.56 kcal mol^−1^ in the NDIH horizontal dimer and −95.49 kcal mol^−1^ in the NDIH vertical dimer.

Most importantly observed in this work, the dispersion term in interactions significantly varies in vertical dimers compared to the horizontal case, and the rate of change is remarkably greater than other interactions as indicated by the dashed box in Fig. 3c and d. The dispersion force energy is nothing but a temporary and appealing induced dipole interaction provided by electrons in adjacent molecules. In our case, it is possible that this force is solely dominated by the radical–radical bondings, due to the fact that the molecules in vertical dimers give rise to such significant change of dispersion energies. After radical–radical bonding, the overlapping region of π electrons is greatly increased and the π–π stacking effect is largely enhanced, which makes the dispersion term in the NDIH vertical dimer reach −57.44 kcal mol^−1^, much larger than the −8.95 kcal mol^−1^ in the NDIH horizontal dimer.

Interaction region indicator (IRI) is an effective method for describing various interaction regions in a system.^47^ According to the interaction intensity, the color of the isosurface map changes. The red area indicates the steric effect, the green area mainly represents the vdW effect with weak attraction, and the blue area indicates strong attraction, such as the covalent bond and hydrogen bond. By IRI analysis, the location and intensity of various interactions in the NDIH vertical dimer are shown by the isosurface map (Fig. 3e). As seen, the green region representing vdW interaction is widely distributed between monomers, which corresponds to most of the exchange and dispersion terms in SAPT calculation and describes radical–radical bonding more specifically in the dimer. Highly directional interaction and a short geometric center distance further demonstrate the strong radical–radical bonding effect and thus the formation of pancake bonding.

The NIR spectra of NDI radical anions at the monomer, dimer and aggregate level were measured experimentally by Penneau *et al.*^48^ For comparison, we conduct Davydov ansatz and TDDFT to simulate the NIR absorption spectra of the NDIH anion vertical dimer. In the experiment, a new band appears around 1140 nm after NDI radical anions form a pancake dimer. In our computation, both calculations show absorption peaks at 1056 or 1134 nm, respectively (Fig. 3f), which qualitatively agree with the experiment and further reveal the characteristic of pancake bonding.^48,49^

**Fig. 4:**
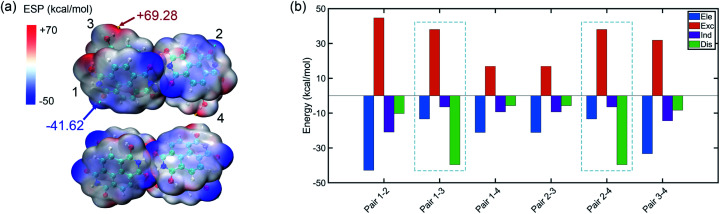
(a) ESP of the NDIH tetramer. Isosurfaces are *ρ* = 0.001 atomic.u. (b) Components of interaction energy in pairs of the NDIH tetramer.

The tetramer is formed from NDIH dimers in horizontal and vertical directions. More details about structure construction, optimization and orbital images are provided in the ESI (Fig. S8 and S9).† The initial vertical stacking of the structure has the lowest energy and is regarded as the NDIH tetramer. We can observe a torsional angle between two layers of monomers, and rotation of monomers at the top layer and bottom layer. ESP can better show the torsional angle (Fig. 4a). Through the proximity of H and O at the end group, the system is twisted, resulting in the overlap of the most positive and negative areas of ESP which reduces the system energy.

For further understanding the interaction in the tetramer, single point calculation at the B3LYP-D3BJ/6-311+G** level with Counterpoise correction and SAPT calculation are also used. Considering the four monomers, the interaction energy by single point calculation is −118.58 kcal mol^−1^. By dividing the monomer of the tetramer into six groups in pairs (the pair numbers correspond to the number in Fig. 4a and Fig. S8†), we obtain the interaction energy in pairs (Table S21†). The difference of interaction energy of a total of six pairs and the tetramer accounts for only 1.18% of the tetramer interaction energy, which proves that the multi-body effect is very weak and can be ignored. Additionally, interaction energies of the six pairs obtained by SAPT are slightly lower than those by single point calculation, but the sequence of interaction energies between the six pairs by the two methods is the same, so the interaction between pairs can still be well described.

Remarkably, similar to NDIH vertical dimers with pancake bonding discussed above, the two adjacent monomers of the upper and lower layers with radical–radical bonding, corresponding to pair 1–3 and pair 2–4, have relatively much stronger intermolecular dispersion −39.59 kcal mol^−1^ than other pairs (Fig. 4b). For example, the dispersion energy is merely −10.23 kcal mol^−1^ for pair 1–2 and −5.69 kcal mol^−1^ for pair 1–4. The essential difference in dispersion further proves the dominant role of radical–radical bonding.

Two monomers in the same layer have larger interaction. For instance, the interaction energy of pair 1–2 is the lowest, in which electrostatic interaction, −42.81 kcal mol^−1^ and exchange interaction, 44.65 kcal mol^−1^ are more important. This is due to the two hydrogen bonds formed between monomers in the same layer, which make the binding closer. The lowest interaction is between the upper and lower layers of non-adjacent monomers. The dispersion effect between them is however very weak, and only one hydrogen bond forms.

**Fig. 5:**
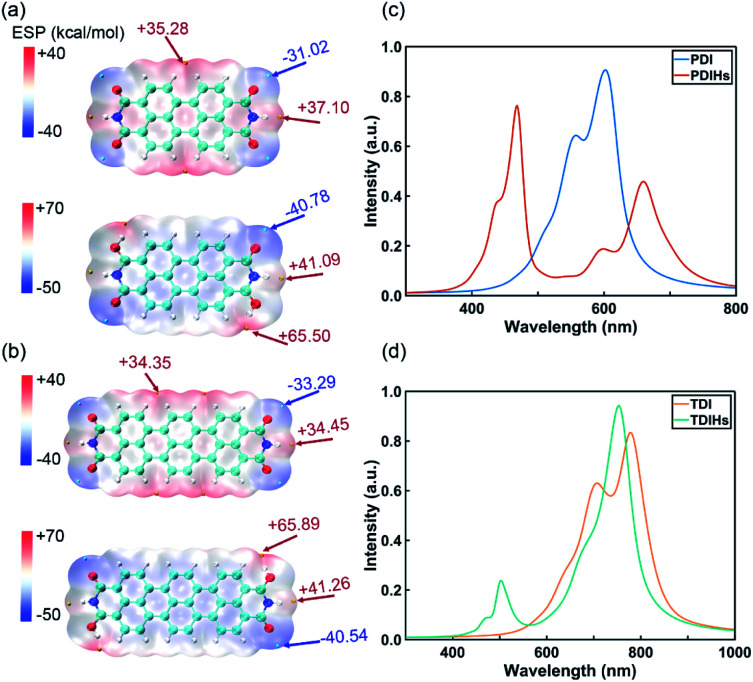
(a) ESP of PDI and PDIHs. (b) ESP of TDI and TDIHs. Isosurfaces are all *ρ* = 0.001 atomic.u. (c) UV-vis absorption spectra of PDI and PDIHs. (d) UV-vis absorption spectra of TDI and TDIHs.

PDI and TDI are two common IPAH materials with poor solubility. They have many structural similarities with NDI. DFT and Davydov ansatz are also used to investigate the properties of PDI, TDI and their radicals. The binding sites of H and charge distributions of PDI and TDI are all similar to those of NDI (Fig. 5a and b). The orbital charge densities of PDI, PDIHs and TDI, TDIHs also resemble those of NDI and NDIHs (Fig. S12†).

The absorption spectra are simulated again with Davydov ansatz (Fig. 5c and d). The main peak and side peak of PDI appear at 602 nm and 557 nm, respectively. PDIHs have a strong peak at 468 nm. Another main peak appears at 660 nm and a side peak appears at 600 nm. The main and side peaks of TDI appear at 777 nm and 707 nm, respectively. Two main absorption peaks of TDIHs appear at 503 nm and 753 nm, respectively. A side peak appears at 474 nm. It is found that with the increase of the number of aromatic rings, the main peaks undergo a redshift.

## Conclusions

In this work, considering the candidates supplying free electrons in experimental hydrazine hydrate solution, we introduced single or double H, H_3_O and NH_4_ in NDI. Through the orbital isosurface map, orbital energy level, Mayer bond order and ESP analysis, we demonstrated that the change of wavefunction induced by radicals and diradicals suggests the formation of a mid-gap polaronic state. The UV-vis absorption spectrum by the variational Davydov ansatz method, in best agreement with the experiment, shows better results than those by TDDFT and FC-TDDFT. The structures and charge variations of the NDIH dimer, and the tetramer are further investigated. Interaction energy is calculated by single point calculation with Counterpoise correction and SAPT. Most importantly, as evidenced by NIR absorption spectra, we find that strong radical–radical pancake bonding forms in π–π stacking aggregates. The attraction from pancake bonding makes the interaction energy of the NDIH vertical dimer much lower than that of the horizontal dimer without obvious π–π stacking. By investigating the properties of IPAHs and their radicals at the level of monomer and aggregate, we deepen the understanding of potential radical-based aggregates, especially the newly found pancake bonding in these materials that may provide references for future experimental studies.

## Data availability

The datasets supporting this article have been uploaded as part of the ESI.†

## Author contributions

Yao Yao, Dehua Hu and Yuguang Ma conceived the concept and directed the work. Jiani Liu performed the DFT studies. Jiarui Zeng designed the relevant code for Davydov ansatz calculation. Jiani Liu performed the Davydov ansatz calculation. Duokai Zhao obtained the experimental spectrum. Jiani Liu wrote the first draft of this manuscript. Jiarui Zeng and Yao Yao modified the manuscript. All authors discussed the results and commented on the manuscript.

## Conflicts of interest

There are no conflicts of interest to declare.

## Supplementary Material

SC-013-D2SC02906E-s001
