# Supplementary material for: Comprehending radicals, diradicals and their bondings in aggregates of imide-fused polycyclic aromatic hydrocarbons
Source: Chem Sci. 2022 Aug 6;13(34):9985–92. doi: 10.1039/d2sc02906e (PMC9430410; doi:10.1039/d2sc02906e)
Supplement: SC-013-D2SC02906E-s001 [file SC-013-D2SC02906E-s001.pdf]

Electronic Supplementary Material (ESI) for Chemical Science.
This journal is © The Royal Society of Chemistry 2022

Electronic Supplementary Information (ESI)

Comprehending radicals, diradicals and their bondings in aggregates of imide-fused polycyclic aromatic hydrocarbons

Jiani Liu, Jiarui Zeng, Duokai Zhao, Yao Yao*, Dehua Hu, Yuguang Ma

Table of Contents

1. Computational Details - 2
2. Experimental Spectrum Characterization - 16
3. Supporting Figures and Tables - 17
4. References - 34

Computational Details

Density functional theory (DFT) calculations of imide-fused polycyclic aromatic hydrocarbons (IPAHs) in this article are all implemented with Gaussian 16 program package.[1] Through DFT calculation, we get electronic structures and essential parameters for Davydov ansatz calculation.[2–4] Multiwfn is a powerful wave function analysis tool.[5] Based on the wavefunction obtained by DFT calculation, it is used to extract molecular orbital file, calculate Mayer bond order, spin density, electrostatic potential (ESP) and interaction region indicator (IRI) in our work.[6–10] Visual Molecular Dynamics (VMD) software is for rendering molecular and aggregate structure images and isosurface maps.[11] In our work, being dissolved and ionized in hydrazine hydrate solution, NDI derivatives show anion radical character. However, after stabilization, the NDI derivatives observed experimentally are neutral clusters and their size is positively correlated with the solution concentration. Based on these points, except the NDIHs anion vertical dimer in NIR spectra calculation, other molecules in this work are at the neutral state. On the other hand, because the solvent composition in computational process had not been precisely acquired, theoretical calculations were all implemented in vacuum. All of the DFT calculation are carried out at ultrafine grid integration.

Functional Test

Energy gap is an essential parameter for Davydov ansatz calculation. The functionals used to calculate energy gap are HSE06, B3PW91 and B3LYP, commonly.[12–16] In this article, these functionals and B3LYP with DFT-D3 dispersion correction are tested to calculate vertical excited energy of NDIHs, respectively.[17] Fig. S1 shows the NDIHs energy gaps of the highest occupied molecular orbital (HOMO) to the lowest unoccupied molecular orbital (LUMO) and HOMO to LUMO+1. The experiment energy gaps are inferred from the two main peak wavelengths in the experimental spectrum. As we can see, energy gaps calculated by B3PW91, B3LYP, B3LYP-D3BJ are all greater than experimental values, while the HSE06 results are more similar to that of experiment. Therefore, in the case of IPAHs monomer, we choose HSE06/6-311G** level to optimize structure and get energy gap. Time-dependent density functional theory (TDDFT) at the level of HSE06/6-311G** is employed for obtaining transition electric dipole moments and oscillator strengths from ground state to excited state. TDDFT and TDDFT with Frank-Condon analyzation (FC-TDDFT) are also employed with the same level to calculation UV-Vis absorption spectrum for comparison with that of Davydov ansatz.

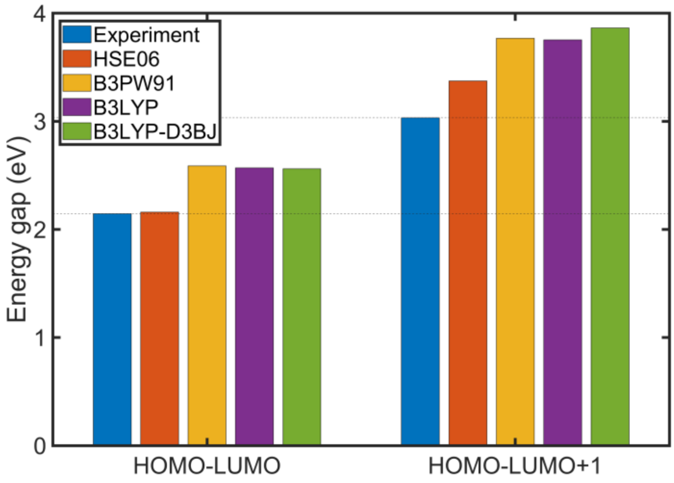

Fig. S1. Energy gaps of HOMO-LUMO and HOMO-LUMO+1 obtained by HSE06, B3PW91, B3LYP and B3LYP-D3BJ with 6-311G**. The energy gaps of experiment are inferred from the main peak wavelengths of two UV-Vis absorption peaks in the experimental spectrum.

In the case of aggregates, dispersion correction needs to be considered. B3LYP-D3BJ/6-311G** level is chosen to optimize structure of aggregates.[18] In the NIR spectrum calculation of NDIHs anion vertical dimer, because the molecule is anion and the charge transfer effect of the excited state need to be considered, ground state calculation is performed at the B3LYP-D3BJ/6-311+G** level for obtaining Davydov ansatz parameters, and TDDFT calculation is performed at the CAM-B3LYP-D3BJ/6-311+G** level.[19–21] All geometries in our system for property calculation are stable points without imaginary frequencies on the potential energy surface.

Hamiltonian

The Hamiltonian of IPAHs can be described as follows:[22–24]

$$\hat{H} = \hat{H}_{el} + \hat{H}_{ph} + \hat{H}_{el-ph} \tag{1}$$

Where $\hat{H}_{el}$, $\hat{H}_{ph}$ and $\hat{H}_{el-ph}$ represent the Hamiltonian of electronic orbitals, nuclear vibrations and electronic-vibrational interactions, respectively. They are defined as,

$$\hat{H}_{el}=\begin{pmatrix}\varepsilon^{(1)} & u_1 & u_2\\ u_1 & \varepsilon^{(2)} & 0\\ u_2 & 0 & \varepsilon^{(3)}\end{pmatrix} \tag{2}$$

$$\hat{H}_{ph}=\sum_q \omega_q \hat{b}_q^{\dagger}\hat{b}_q \tag{3}$$

$$\hat{H}_{el-ph}=\sum_q \begin{pmatrix}\kappa_q^{1\,(1)} & 0 & 0\\ 0 & \kappa_q^{1\,(2)} & 0\\ 0 & 0 & \kappa_q^{1\,(3)}\end{pmatrix}\left(\hat{b}_q^{\dagger}+\hat{b}_q\right)+\sum_q \begin{pmatrix}\kappa_q^{2\,(1)} & 0 & 0\\ 0 & \kappa_q^{2\,(2)} & 0\\ 0 & 0 & \kappa_q^{3\,(3)}\end{pmatrix}\left(\hat{b}_q^{\dagger}+\hat{b}_q\right)^2 \tag{4}$$

where $\varepsilon^{(1)}$, $\varepsilon^{(2)}$ and $\varepsilon^{(3)}$ represent the vertical energies of HOMO, LUMO and LUMO+1, respectively. u_1 (u_2) is the effective coupling between HOMO and LUMO (HOMO and LUMO+1). ω_q is the frequency corresponding to the q-th vibrational mode. $\hat{b}_q^{\dagger}\left(\hat{b}_q\right)$ is the creation (annihilation) operator. $\kappa_q^{1\,(n)}$ is the first-order electronic-vibrational coupling strength and $\kappa_q^{2\,(n)}$ is the second-order coupling strength, which can be calculated according to

$$\kappa_q^{1\,(n)}=\sqrt{\frac{\hbar^2}{2\bar{m}_q\omega_q}}p_q^{1\,(n)} \tag{5}$$

$$\kappa_q^{2\,(n)}=\frac{\hbar^2}{2\bar{m}_q\omega_q}p_q^{2\,(n)} \tag{6}$$

In this article, we find the maximum shift of all atoms moving in three directions in all vibration modes at Cartesian coordinates, and limit the maximum shift in this direction to no more than -0.02, - 0.01, 0.01 and 0.02 Å respectively. We set that all atoms in all modes change in equal proportion and get structures moved by four distances. Then, we use the quadratic polynomial to fit the energy differences before and after the moving atoms. $p_q^{0\,(n)}$ is the fitted constant term, which is always 0, as it is the energy difference at equilibrium geometry. $p_q^{1\,(n)}$ and $p_q^{2\,(n)}$ are the first-order and second-order coupling coefficients at the n-th energy level of the q-th vibration mode respectively. $\bar{m}_q$ is the effective molecular mass, which can be calculated as

$$\bar{m}_q=\frac{\sum_s m_s r_{q,s}^2}{\sum_s r_{q,s}^2} \tag{7}$$

Where, $r_{q,s}$ represents the shift of the s-th atom of the q-th vibrational mode. m_s is the mass of the s-th atom.

Expression of Davydov Ansatz and Observable Quantities

In this work, we adopt the variational Davydov ansatz to study the excitation dynamics and obtain UV-Vis absorption spectra of IPAHs, which has been shown its advantages in simulating the quantum dynamics in numbers of chemical and physical domain.[25–30] Considering three electronic orbitals, the vibrations of the nuclei, and their couplings up to second-order truncation, the multi-D2 method is taken into account in our calculations, which can be written as

$$|D_2^M(t)\rangle=\sum_i^M A_i(t)|1\rangle|f_i(t)\rangle_{ph}+B_i(t)|2\rangle|f_i(t)\rangle_{ph}+C_i(t)|3\rangle|f_i(t)\rangle_{ph} \tag{8}$$

Where $|1\rangle$, $|2\rangle$ and $|3\rangle$ represent three diabatic electronic orbitals, respectively. $A_i(t)$, $B_i(t)$ and $C_i(t)$ are time-dependent normalization coefficients. $|f_i(t)\rangle_{ph}$ is in the coherent-state representation, which is written as

$$|f_i(t)\rangle_{ph}=\exp\left(\sum_q f_{i,q}(t)b_q^{\dagger}-h.c.\right)|0\rangle_{ph} \tag{9}$$

where $f_{i,q}(t)$ is the variational parameter of the coherent states in different electronic orbitals, and $h.c.$ represents the Hermitian conjugate. All of variational parameters, $A_i(t)$, $B_i(t)$, $C_i(t)$ and $f_{i,q}(t)$ are obtained by the Dirac-Frenkel time-dependent variational principle:

$$\frac{d}{dt}\left(\frac{\partial L}{\partial\dot{\alpha}^*}\right)-\frac{\partial L}{\partial\alpha^*}=0 \tag{10}$$

In which α is the variational parameters, $\dot{\alpha}$ is the time derivative of α . The Lagrangian L is given as

$$L=\left\langle D_2^M(t)\right|\frac{i\hbar}{2}\frac{\overleftarrow{\partial}}{\partial t}-\frac{i\hbar}{2}\frac{\overrightarrow{\partial}}{\partial t}-\hat{H}\left|D_2^M(t)\right\rangle \tag{11}$$

The absorption spectrum can be obtained by

$$I(\omega)=\int_{-\infty}^{\infty} dt\, e^{i\omega t}\left\langle D_2^M(0)\right|\exp\left(\frac{-i\hat{H}t}{\hbar}\right)\left|D_2^M(0)\right\rangle \tag{12}$$

We use $W(t)$ to express the inner product in the integral:

$$\begin{aligned} W(t) &= \left\langle D_2^M(0)\right|\exp\left(\frac{-i\hat{H}t}{\hbar}\right)\left|D_2^M(0)\right\rangle \\ &= \left\langle D_2^M(t/2)\middle|D_2^M(t/2)\right\rangle \end{aligned} \tag{13}$$

$W(t)$ is called autocorrelation function which is the overlap between initial and final states. Then we can get spectral lines with the Fourier transformation of the autocorrelation function. In practical experiment, lines of the spectra are broaden caused by the resolution of the spectrometer.[30] In order to reproduce this effect, here we multiply $W(t)$ with a phenomenological damping curve $g(t)=\exp(-t/\tau)$ for Lorentzian broadening, where τ is a model-dependent damping time.

Parameters for Davydov Ansatz Calculation

By DFT calculation, we obtain frontier orbital energies below:

Table S1. Orbital energies (eV) of HOMO, LUMO and LUMO+1 of NDI, NDIH-diradical, $NDIH_3O$-diradical and $NDINH_4$-diradical.

	HOMO	LUMO	LUMO+1
NDI	-7.277	-4.005	-2.239
NDIH-diradical[a]	-4.610	-2.463	-1.129
$NDIH_3O$-diradical[b]	-4.092	-1.924	-0.657
$NDINH_4$-diradical[c]	-4.047	-1.879	-0.643

[a] NDIH-diradical represents NDI with double introduced H. [b] $NDIH_3O$-diradical represents NDI with double introduced H_3O. [c] $NDINH_4$-diradical represents NDI with double introduced NH_4.

Table S2. Orbital energies (eV) of HOMO, SOMO and LUMO of NDIH-radical, $NDIH_3O$-radical, $NDINH_4$-radical.

	HOMO	SOMO[a]	LUMO	LUMO+1
NDIH-radical[b]	-7.146	-4.980	-2.497	-1.609
$NDIH_3O$-radical[c]	-6.787	-4.571	-2.133	-1.263
$NDINH_4$-radical[d]	-6.801	-4.579	-2.139	-1.307

[a] SOMO appears in α orbitals, we only consider energy of α orbitals here. [b] NDIH-radical represents NDI with single introduced H. [c] $NDIH_3O$-radical represents NDI with single introduced H_3O. [d] $NDINH_4$-radical represents NDI with single introduced NH_4.

Table S3. Orbital energies (eV) of HOMO, LUMO and LUMO+1 of PDI, PDIHs, TDI, TDIHs.

	HOMO	LUMO	LUMO+1
PDI	-6.189	-4.056	-2.424
PDIHs[a]	-4.493	-2.597	-1.790
TDI	-5.645	-4.071	-2.848
TDIHs[b]	-4.423	-2.768	-1.986

[a] PDIHs represents PDI with double introduced H. [b] TDIHs represents TDI with double introduced H.

Considering that multiple IPAHs systems need to be calculate, and the vibration modes of NDI with the minimum atomic numbers reach 78, it is necessary to neglect the weak coupling mode to simplify the calculation. We only take the mode with $\left|\kappa_q^{1(n)}(\kappa_q^{2(n)})/\omega_q\right|>0.05$ for calculation, and the corresponding frequency and coupling strength are as follows:

Table S4. Frequency (ω_q), the first-order electronic-vibrational coupling strength of HOMO ($\kappa^{1(n)}_{q-HOMO}$), LUMO ($\kappa^{1(n)}_{q-LUMO}$) and LUMO+1 ($\kappa^{1(n)}_{q-LUMO+1}$) and the second-order coupling strength of HOMO ($\kappa^{2(n)}_{q-HOMO}$), LUMO ($\kappa^{2(n)}_{q-LUMO}$) and LUMO+1 ($\kappa^{2(n)}_{q-LUMO+1}$), for NDI.

NDI						
ω_q (eV)	$\kappa^{1(n)}_{q-HOMO}$ (eV)	$\kappa^{1(n)}_{q-LUMO}$ (eV)	$\kappa^{1(n)}_{q-LUMO+1}$ (eV)	$\kappa^{2(n)}_{q-HOMO}$ (eV)	$\kappa^{2(n)}_{q-LUMO}$ (eV)	$\kappa^{2(n)}_{q-LUMO+1}$ (eV)
0.006976	0.000000	0.000000	0.000000	0.000000	0.000000	0.003126
0.017839	0.000000	0.000000	0.000000	0.000000	0.002084	0.004167
0.018431	0.000000	0.000000	0.000000	0.000000	0.000000	0.004492
0.024351	0.000000	0.000000	0.000000	-0.002761	0.000000	0.002761
0.027055	0.000160	0.000000	0.000107	-0.001424	-0.000475	-0.002849
0.030372	0.000000	0.000000	0.000000	0.000000	0.000000	0.001583
0.038891	-0.015828	0.004744	-0.013603	0.000294	0.000588	0.000881
0.050051	0.000155	0.000104	0.000155	-0.000894	-0.002682	-0.002235
0.052636	0.022642	0.032780	0.004140	0.001487	0.001487	0.002082
0.056271	0.000000	0.000000	0.000000	-0.000911	0.003646	0.003646
0.058200	0.000000	0.000000	0.000000	-0.003183	-0.003183	0.001591
0.066766	0.000000	0.000000	0.000000	-0.003690	0.000000	0.003690
0.069786	-0.004264	0.019169	0.009620	0.000000	0.000621	0.001035
0.082995	0.000000	0.000000	0.000000	-0.003533	-0.001766	0.005299
0.087718	0.038439	0.016601	0.006626	-0.001058	0.000000	-0.000423
0.089378	0.000000	0.000000	0.000000	-0.002627	0.000000	0.007881
0.092797	0.000000	0.000000	0.000000	-0.001651	-0.001651	0.008254
0.101336	0.000000	0.000000	0.000000	-0.001629	0.000000	0.006109
0.130549	0.010768	-0.030463	-0.018514	-0.000744	0.001115	0.000000
0.141862	-0.004730	0.010659	0.011497	-0.000598	-0.001195	-0.001793
0.159080	-0.000284	0.000406	0.000284	0.000824	0.001099	0.008241
0.167890	0.039583	0.031256	0.012942	0.000433	0.001516	0.001949
0.177202	-0.055440	-0.014341	-0.093413	0.000130	0.000259	-0.000130
0.181586	-0.051283	0.087933	-0.026992	0.001014	-0.000422	-0.002956
0.206442	0.048316	-0.100224	-0.145577	0.000726	-0.000635	0.000091
0.208476	0.000107	0.000086	0.000279	-0.012255	0.002374	0.002681
0.225477	0.043501	0.083921	0.090672	-0.002221	-0.000688	-0.000899
0.447542	0.011181	0.010385	0.023378	0.000000	0.000326	0.000326

Table S5. Frequency (ω_q), the first-order electronic-vibrational coupling strength of HOMO ($\kappa^{1(n)}_{q-HOMO}$), LUMO ($\kappa^{1(n)}_{q-LUMO}$) and LUMO+1 ($\kappa^{1(n)}_{q-LUMO+1}$) and the second-order coupling strength of HOMO ($\kappa^{2(n)}_{q-HOMO}$), LUMO ($\kappa^{2(n)}_{q-LUMO}$) and LUMO+1 ($\kappa^{2(n)}_{q-LUMO+1}$), for NDIH-radical.

NDIH-radical						
ω_q (eV)	$\kappa^{1(n)}_{q-HOMO}$ (eV)	$\kappa^{1(n)}_{q-LUMO}$ (eV)	$\kappa^{1(n)}_{q-LUMO+1}$ (eV)	$\kappa^{2(n)}_{q-HOMO}$ (eV)	$\kappa^{2(n)}_{q-LUMO}$ (eV)	$\kappa^{2(n)}_{q-LUMO+1}$ (eV)
0.009547	0.000000	0.000000	0.000000	0.000000	0.000000	0.010387
0.012689	0.000000	0.000000	-0.000161	0.000000	0.000000	0.004297
0.018662	0.000000	0.000000	0.000000	0.000000	0.004364	0.000000
0.021223	0.000000	0.000000	0.000000	0.000000	0.004902	0.000000
0.024803	0.000000	0.000000	0.000000	0.000000	0.000000	0.009169
0.026816	0.008978	-0.009506	0.005457	0.002582	0.000000	0.001291
0.030076	0.000000	0.000000	0.000000	0.000000	0.004107	0.004107
0.035080	0.000000	0.000000	0.000000	0.000000	0.005539	0.000000
0.037567	0.000000	0.000000	0.000000	0.009933	0.009933	0.009933
0.038146	-0.001948	0.008243	0.008917	0.005615	0.005615	-0.000936
0.041248	-0.000687	-0.006642	-0.009161	0.002914	0.004856	0.001943
0.045410	-0.007691	-0.010811	-0.011609	0.002632	0.003510	0.004387
0.050382	-0.015799	-0.007752	0.012329	0.008176	0.006359	0.002725
0.051465	0.012846	-0.002892	0.003228	0.000000	0.000754	-0.000754
0.051963	0.000000	0.000000	0.000000	0.005506	0.002753	0.002753
0.055333	-0.001353	0.000800	-0.004429	-0.004415	-0.003784	0.003154
0.060336	-0.006501	-0.006239	0.001051	-0.000719	0.000000	-0.001437
0.061774	0.000000	0.000000	0.000000	-0.003120	0.000000	0.000000
0.065908	0.000000	0.000000	0.000000	0.000000	0.004134	0.002067
0.069282	-0.021765	-0.018957	-0.026032	0.002431	0.001458	0.000000
0.073275	0.000000	0.000000	0.000075	0.003706	0.001853	0.006486
0.075523	0.002416	0.001717	-0.000890	0.004043	0.002695	0.005391
0.081507	0.006180	0.016575	0.018250	0.001668	0.000556	0.000000
0.082979	-0.001414	0.015490	0.010372	0.000000	0.000000	-0.002268
0.083084	-0.000102	-0.000510	0.000102	0.005210	0.008684	0.010421
0.087142	0.009656	0.003697	0.017049	-0.001015	0.000000	-0.000507
0.087212	0.000000	-0.000765	0.000127	0.000000	0.005413	0.002707
0.094539	0.000686	0.006004	0.011265	0.000000	-0.001635	0.000000
0.095532	-0.005908	-0.006052	-0.019236	0.001730	0.000000	0.002595
0.103161	0.012465	0.005069	-0.004294	0.001186	0.000000	0.002371
0.115756	-0.006901	-0.019681	-0.004984	0.000681	-0.000681	0.001361
0.121039	0.000000	0.000000	0.000000	0.004279	0.004279	0.008559
0.130236	-0.011607	0.001912	0.001502	0.000777	-0.000777	-0.000777
0.138208	-0.032694	-0.004901	-0.005132	-0.000554	-0.001662	-0.004987
0.140796	-0.016181	0.021431	-0.033309	0.003704	-0.004939	0.007408
0.145152	0.015238	0.006571	-0.009714	0.004535	-0.001512	0.012093
0.147564	-0.017211	-0.003953	-0.010870	0.003391	0.000000	0.000000
0.147632	-0.002417	-0.011192	0.000537	0.000000	0.008016	0.001336
0.149612	0.006995	0.012090	-0.003713	0.003729	0.000000	0.001243
0.154531	0.003338	0.039753	0.037249	-0.000959	0.001918	-0.002878
0.158537	-0.030313	0.022920	0.014616	0.002695	0.001078	-0.003774
0.160124	0.032768	-0.066288	0.053425	0.005877	-0.002204	0.002571
0.165476	0.022640	0.003852	-0.024055	-0.007210	-0.008240	-0.002060
0.168421	-0.017554	-0.006109	0.004565	0.003446	0.001378	0.003446
0.172710	0.048540	0.003277	0.000555	0.001542	-0.000514	0.000000
0.174815	0.044376	-0.027116	-0.014938	-0.001599	-0.004478	0.005117
0.176030	-0.010335	0.004251	0.036429	-0.000895	-0.000895	0.004477
0.178230	0.033337	-0.033201	0.014333	-0.000686	0.001714	0.014743
0.181529	-0.008859	-0.054102	-0.024226	0.002043	0.002724	0.000340
0.184284	0.018284	0.004312	-0.018514	0.001653	0.000000	0.000551
0.189467	-0.045522	0.001044	-0.009399	0.001127	-0.003004	-0.001878
0.194351	0.004601	0.048714	0.026193	0.000979	0.000326	0.021860
0.198687	0.040922	0.010348	0.023317	-0.000731	0.000731	0.000000
0.200070	0.025569	-0.011242	-0.049839	0.000275	0.000824	0.002196
0.201976	0.019254	0.018374	0.046493	-0.000382	0.004771	-0.001717
0.205337	0.078783	0.116789	-0.039282	0.000222	0.000665	-0.001995
0.220310	-0.027630	-0.017854	-0.061118	-0.000317	-0.000475	-0.000634
0.222228	-0.014257	-0.032001	-0.017837	0.000162	0.000485	-0.000162
0.223224	-0.067364	-0.090663	-0.083426	-0.001554	-0.000311	-0.002487
0.447963	0.012687	0.022758	0.007063	0.000000	0.000713	0.000713

Table S6. Frequency (ω_q), the first-order electronic-vibrational coupling strength of HOMO ($\kappa^{1(n)}_{q-HOMO}$), LUMO ($\kappa^{1(n)}_{q-LUMO}$) and LUMO+1 ($\kappa^{1(n)}_{q-LUMO+1}$) and the second-order coupling strength of HOMO ($\kappa^{2(n)}_{q-HOMO}$), LUMO ($\kappa^{2(n)}_{q-LUMO}$) and LUMO+1 ($\kappa^{2(n)}_{q-LUMO+1}$), for NDIH-diradical.

NDIH-diradical						
ω_q (eV)	$\kappa^{1(n)}_{q-HOMO}$ (eV)	$\kappa^{1(n)}_{q-LUMO}$ (eV)	$\kappa^{1(n)}_{q-LUMO+1}$ (eV)	$\kappa^{2(n)}_{q-HOMO}$ (eV)	$\kappa^{2(n)}_{q-LUMO}$ (eV)	$\kappa^{2(n)}_{q-LUMO+1}$ (eV)
0.020624	0.000000	0.000000	0.000000	0.002866	0.002866	0.002866
0.022144	0.000000	0.000000	0.000000	0.000000	0.003127	0.003127
0.026441	0.000068	-0.000068	0.000136	-0.003858	-0.002315	-0.002315
0.030163	0.000000	0.000000	0.000000	0.000000	0.002112	0.000000
0.032807	0.000000	0.000000	0.000000	0.000000	0.002113	0.004225
0.034333	0.000000	0.000000	0.000000	0.000000	0.006715	0.006715
0.037418	0.002668	-0.007198	-0.004406	0.001925	0.000642	-0.000642
0.038212	0.000000	0.000000	0.000000	0.000000	0.004988	0.004988
0.040852	-0.002043	0.012371	0.013586	-0.000508	0.002033	0.000000
0.044130	-0.000058	-0.000058	-0.000058	-0.000553	-0.001660	-0.003874
0.048213	0.000000	0.000000	0.000000	0.004010	0.004010	0.002005
0.049189	0.010759	-0.006956	-0.016614	0.000835	0.001252	0.001669
0.052556	0.003916	-0.017388	0.000888	-0.000454	-0.002272	0.000909
0.060173	0.000147	0.000098	0.000245	0.001604	0.001604	0.000401
0.066095	0.000000	0.000000	0.000000	-0.001344	0.000000	0.004032
0.068949	-0.029439	-0.019400	-0.029917	0.000264	0.000793	0.001851
0.072007	0.000000	0.000000	0.000000	0.001998	0.002997	0.005994
0.075314	0.011012	0.003686	0.007562	-0.001117	0.000745	0.001117
0.081152	0.000000	0.000000	0.000000	0.001274	0.006370	0.003822
0.083676	0.000000	0.000000	0.000000	0.002227	0.006682	0.004455
0.085520	0.000000	0.000000	0.000000	0.000000	0.003366	0.005049
0.086466	0.009495	0.003940	0.020330	0.000776	0.001035	0.000000
0.088682	0.000000	0.000000	0.000000	0.000000	0.008068	0.004034
0.093309	0.000000	0.000000	0.000000	0.002348	0.004697	0.007045
0.094047	0.000000	0.000000	0.000000	0.000000	0.005598	0.005598
0.094195	0.006434	0.018016	0.011421	0.001438	0.001438	-0.000479
0.094781	0.000000	0.000000	0.000000	0.002781	0.002781	0.005561
0.100588	0.000000	0.000000	0.000000	0.001465	0.003662	0.006591
0.101352	0.000000	0.000000	0.000000	0.000000	0.003589	0.005384
0.119571	0.000000	0.000000	0.000000	0.002260	0.002260	0.006779
0.119659	0.000000	0.000000	0.000000	0.002275	0.002275	0.006826
0.130337	-0.018612	0.018058	0.000785	0.000000	0.001066	-0.000355
0.137241	-0.025038	-0.041346	0.005277	-0.000525	-0.000262	0.000787
0.145113	-0.023581	-0.017883	-0.020817	0.000000	0.003183	0.000530
0.146916	-0.001882	-0.022440	0.018189	-0.003238	-0.004047	0.000809
0.151314	0.024737	0.029240	0.045100	0.001420	0.000710	0.004970
0.160023	0.019824	0.013100	-0.025389	0.001742	0.001493	0.000249
0.165515	0.038043	-0.009204	-0.022926	-0.001556	-0.001556	-0.003630
0.168047	0.002203	-0.027061	0.010600	0.000000	0.000576	-0.001152
0.175093	0.000173	0.000260	0.000173	0.000000	0.000312	0.010624
0.177024	0.062472	-0.047788	-0.036942	0.000742	-0.000186	-0.000557
0.180993	-0.030068	0.028572	0.042424	0.002329	0.000000	0.001941
0.192709	0.026632	0.015289	0.000174	-0.000140	-0.000140	-0.000421
0.195693	-0.000066	-0.000066	-0.000066	-0.001100	-0.001100	0.017050
0.199751	-0.063156	-0.039357	-0.061855	-0.000480	-0.000640	-0.001761
0.204115	-0.010649	0.059923	-0.035023	0.000000	0.001267	-0.001037
0.205236	0.000053	0.000027	0.000027	-0.000590	0.000000	0.011332
0.210945	0.077127	0.088189	-0.044705	-0.000216	0.001940	-0.003557
0.221978	0.080375	0.099329	0.094523	-0.002546	-0.000933	-0.003649
0.391600	0.007696	0.005611	0.008540	-0.000411	0.000000	0.001233
0.448096	0.013860	0.027673	0.012600	0.000726	0.000363	0.000000

Table S7. Frequency (ω_q), the first-order electronic-vibrational coupling strength of HOMO ($\kappa^{1(n)}_{q-HOMO}$), LUMO ($\kappa^{1(n)}_{q-LUMO}$) and LUMO+1 ($\kappa^{1(n)}_{q-LUMO+1}$) and the second-order coupling strength of HOMO ($\kappa^{2(n)}_{q-HOMO}$), LUMO ($\kappa^{2(n)}_{q-LUMO}$) and LUMO+1 ($\kappa^{2(n)}_{q-LUMO+1}$), for $NDIH_3O$-diradical.

$NDIH_3O$-diradical						
ω_q (eV)	$\kappa^{1(n)}_{q-HOMO}$ (eV)	$\kappa^{1(n)}_{q-LUMO}$ (eV)	$\kappa^{1(n)}_{q-LUMO+1}$ (eV)	$\kappa^{2(n)}_{q-HOMO}$ (eV)	$\kappa^{2(n)}_{q-LUMO}$ (eV)	$\kappa^{2(n)}_{q-LUMO+1}$ (eV)
0.008904	0.000000	0.000000	0.000000	0.004987	0.004987	0.004987
0.009324	0.000000	0.000000	0.000000	0.005338	0.000000	0.005338
0.011327	0.001333	0.001333	0.000267	0.000000	0.000000	-0.002961
0.012705	-0.005187	-0.006452	-0.007211	0.002667	0.002667	0.008002
0.013270	0.000000	0.000000	0.000000	0.008909	0.000000	0.008909
0.017489	0.000000	0.000000	0.000000	0.015460	0.000000	0.000000
0.021297	0.000000	0.000000	0.000000	0.000000	0.005311	0.005311
0.021708	-0.000445	-0.000267	-0.000445	0.005270	0.009223	0.006588
0.022109	0.019792	0.019440	0.018032	0.005158	0.003869	0.003869
0.024304	0.000000	0.000000	0.000000	0.001730	0.001730	0.003459
0.028995	0.000350	0.000210	-0.000280	0.001631	0.000815	0.003261
0.030260	0.000000	0.000000	0.000000	0.000000	0.002350	0.002350
0.031624	0.000000	0.000000	0.000000	-0.002305	0.002305	0.002305
0.033934	-0.003358	-0.002719	-0.002399	0.012788	0.004263	-0.004263
0.033977	0.000000	0.000000	0.000000	0.000000	0.003008	0.003008
0.034531	-0.065458	-0.067661	-0.067189	-0.008253	0.000000	0.000000
0.037846	0.000000	0.000000	0.000000	0.000000	0.008554	0.008554
0.038553	0.000000	0.000000	0.000000	0.000000	0.007395	0.007395
0.039440	0.011551	-0.004596	-0.005503	0.001219	0.001219	0.002438
0.041752	-0.006914	-0.012635	-0.012933	0.000592	0.000000	0.002960
0.047721	0.000000	0.000000	0.000000	0.004516	0.003011	0.003011
0.051198	0.020889	0.000406	-0.006439	0.000428	0.001714	0.001714
0.052777	-0.004205	-0.022089	-0.002395	0.000000	0.000000	-0.001889
0.066795	0.000000	0.000000	0.000000	-0.001241	0.001241	0.003724
0.069743	-0.024516	-0.014888	-0.025932	-0.000546	-0.001364	0.001091
0.072291	0.000000	0.000000	0.000000	0.000978	0.002935	0.005870
0.076044	0.012492	0.005670	0.009910	0.001416	0.001771	0.001416
0.082248	0.000000	0.000000	0.000000	0.000000	0.006276	0.003138
0.085752	0.000000	0.000000	0.000000	0.001748	0.006992	0.005244
0.087138	0.005790	-0.001418	0.017174	-0.000776	0.000259	0.000259
0.089856	0.000000	0.000000	0.000000	0.000000	0.006142	0.004607
0.094694	0.000000	0.000000	0.000000	0.000000	0.004869	0.004869
0.095538	0.004549	0.015895	0.010222	0.001910	0.002387	0.002387
0.097055	0.000000	0.000000	0.000000	0.002100	0.004201	0.006301
0.104802	0.000000	0.000000	0.000000	0.001929	0.001929	0.005788
0.122851	0.000000	0.000000	0.000000	0.002311	0.002311	0.006932
0.122999	0.000000	0.000000	0.000000	0.002315	0.002315	0.006944
0.130605	-0.021168	0.015249	0.002510	-0.000748	-0.001869	-0.000748
0.138262	-0.021485	-0.035225	0.005545	-0.000277	-0.000277	-0.001385
0.146125	0.012598	-0.002313	0.017650	0.002469	0.002469	-0.000617
0.147266	-0.006927	-0.027707	0.006469	0.000712	0.001423	-0.002135
0.153942	0.035875	0.034891	0.056731	-0.001434	-0.003585	0.000717
0.160571	0.021518	0.020849	-0.018656	0.002302	0.002072	-0.001841
0.165976	0.030325	-0.014240	-0.028596	0.000554	0.000554	0.000554
0.168658	0.000789	-0.026229	0.012062	0.000641	0.001283	-0.001603
0.174665	-0.000309	-0.000177	0.000088	0.000326	-0.000326	0.009445
0.176245	0.068657	-0.043868	-0.039012	-0.000497	-0.001160	-0.001492
0.181137	0.023953	-0.023049	-0.038516	0.001261	-0.001261	0.002522
0.191844	0.030187	0.029833	-0.000265	0.000724	0.000869	0.000724
0.195538	-0.000382	-0.000347	-0.000278	-0.000602	-0.001204	0.015849
0.200160	-0.066012	-0.046249	-0.066106	-0.000329	0.000658	-0.000987
0.202012	0.000710	0.044750	-0.021250	-0.001752	0.000000	-0.001168
0.203232	0.003773	-0.058950	0.028232	-0.000657	0.000985	-0.001314
0.204346	0.000000	-0.000030	-0.000030	-0.000727	0.000727	0.014388
0.210457	-0.078209	-0.081979	0.040568	0.000347	0.002548	-0.003590
0.220797	-0.081310	-0.099832	-0.092082	-0.002394	-0.000532	-0.003370
0.441406	0.043437	0.047243	0.038346	-0.000754	-0.000754	-0.000754
0.448565	-0.012794	-0.026810	-0.012135	-0.000369	0.000369	0.000369

Table S8. Frequency (ω_q), the first-order electronic-vibrational coupling strength of HOMO ($\kappa_{q-HOMO}^{1(n)}$), LUMO ($\kappa_{q-LUMO}^{1(n)}$) and LUMO+1 ($\kappa_{q-LUMO+1}^{1(n)}$) and the second-order coupling strength of HOMO ($\kappa_{q-HOMO}^{2(n)}$), LUMO ($\kappa_{q-LUMO}^{2(n)}$) and LUMO+1 ($\kappa_{q-LUMO+1}^{2(n)}$), for $NDINH_4$-diradical.

NDINH4-diradical						
ω_q (eV)	$\kappa_{q-HOMO}^{1(n)}$ (eV)	$\kappa_{q-LUMO}^{1(n)}$ (eV)	$\kappa_{q-LUMO+1}^{1(n)}$ (eV)	$\kappa_{q-HOMO}^{2(n)}$ (eV)	$\kappa_{q-LUMO}^{2(n)}$ (eV)	$\kappa_{q-LUMO+1}^{2(n)}$ (eV)
0.003266	0.000270	0.000000	-0.000270	0.036325	0.024217	0.060542
0.004468	0.022549	0.023470	0.021859	0.017648	-0.008824	0.026472
0.006948	0.000000	0.000337	0.000000	-0.009462	0.014192	0.000000
0.008798	-0.000205	-0.000205	-0.000205	-0.001760	0.001760	0.005279
0.008961	0.006721	0.004537	0.008233	0.000000	-0.018820	-0.004705
0.010369	0.001051	0.000000	0.000526	0.046045	-0.023022	0.057556
0.010732	-0.004663	-0.002332	-0.001295	-0.011187	0.022374	0.011187
0.013235	0.000000	0.000000	0.000000	0.006343	-0.006343	0.006343
0.017471	-0.010549	-0.012816	-0.007197	-0.001620	-0.004860	0.000000
0.021062	0.000000	-0.000170	0.000000	-0.002409	-0.001205	0.002409
0.023481	0.000949	-0.001314	0.000219	0.000889	-0.000889	0.000889
0.024626	0.000069	0.000069	0.000069	-0.000791	0.000791	0.003957
0.029625	-0.020774	-0.022219	-0.017335	0.000000	-0.002366	0.001577
0.031038	0.012782	0.008116	0.006290	0.005336	0.006861	0.001525
0.031522	0.000000	0.000000	0.000000	0.000000	0.001786	0.001786
0.036352	0.000000	0.000000	0.000000	0.000000	0.003164	0.001582
0.038916	0.026998	0.038588	0.036712	0.003740	0.001496	0.005236
0.040508	0.005775	-0.005338	-0.004550	-0.005105	-0.011486	-0.002552
0.040955	0.000133	0.000266	0.000000	0.000000	0.002942	0.000000
0.041164	-0.016009	-0.008585	-0.005917	0.004486	0.004486	-0.004486
0.044655	0.000075	0.000075	0.000075	0.002837	0.002837	0.000946
0.047878	-0.012976	-0.007316	-0.010284	0.003970	0.003970	0.002382
0.050107	0.034375	0.016981	0.009348	0.002334	0.002917	0.002917
0.052356	0.003000	0.019426	0.001328	0.000403	0.001209	-0.000403
0.053086	0.000000	0.000000	-0.000094	0.001461	0.004383	0.004383
0.055629	-0.006489	-0.018919	-0.018279	0.002784	0.001392	0.000000
0.058101	0.000159	0.000106	0.000053	0.001399	0.001399	0.005597
0.067583	-0.004871	0.001873	-0.003211	-0.001432	0.000955	0.001432
0.069338	-0.024317	-0.013705	-0.023561	-0.001078	-0.001510	-0.001941
0.073782	0.000045	0.000045	0.000045	0.001718	0.003779	0.004466
0.076342	0.007020	0.001635	0.004467	-0.000796	0.000530	0.000530
0.083294	-0.000490	-0.004227	0.007720	-0.000626	0.003754	0.001251
0.086899	-0.003407	-0.000473	-0.015427	0.001866	0.001493	0.001493
0.087060	-0.007016	-0.000324	-0.008096	0.000000	0.002913	0.002913
0.088079	0.000000	0.000137	-0.000069	-0.001567	0.005484	0.003917
0.091356	0.000122	0.000182	0.000122	0.003076	0.009845	0.004307
0.095006	0.002506	-0.005611	-0.001580	-0.000989	0.001978	0.001978
0.096456	0.000886	0.011879	0.003334	0.001357	0.004072	0.002715
0.098906	-0.007305	-0.005566	-0.002725	0.003361	0.002801	0.004482
0.103372	0.005895	0.002895	0.003948	0.001847	0.004156	0.006003
0.123955	0.006992	0.016812	0.001257	-0.001029	0.000000	-0.006172
0.131393	0.018937	-0.012948	-0.009279	-0.001186	-0.002372	-0.000296
0.138148	0.019125	0.036292	-0.007454	-0.000945	-0.000945	-0.000472
0.144359	0.006094	-0.005857	0.012187	-0.001750	-0.001167	-0.001750
0.144888	-0.022658	-0.017827	-0.023610	0.002315	-0.002315	-0.003086
0.147294	0.013920	0.034663	0.002311	0.000000	-0.000505	0.000505
0.159573	0.022083	0.018625	-0.021496	0.001241	0.000177	-0.003015
0.161005	0.035948	0.013749	0.025541	0.000760	0.000380	0.001519
0.168246	-0.005334	0.031752	-0.008129	-0.000658	-0.000219	-0.002414
0.171324	0.000000	0.000092	0.000183	0.000701	-0.001051	0.009807
0.174260	-0.064507	0.038630	0.063077	-0.001290	-0.002418	-0.001290
0.179376	-0.004168	0.004717	0.038885	0.001398	0.002447	0.000699
0.182311	-0.035778	0.035376	0.000151	0.000420	0.001259	0.000839
0.192991	0.033604	0.020874	0.012867	-0.000195	-0.000781	-0.000781
0.195892	0.000035	-0.000035	-0.000035	-0.001396	-0.000997	0.013361
0.199563	-0.065148	-0.057553	-0.057609	-0.000264	0.000396	-0.000659
0.202391	-0.008320	0.066457	-0.040722	-0.000356	0.001067	-0.001067
0.203604	-0.000133	-0.000133	-0.000027	-0.000118	0.001302	0.014321
0.207507	-0.010511	-0.007929	-0.003627	-0.000630	-0.001889	0.000000
0.209872	-0.076775	-0.080548	0.034027	0.000100	0.002601	-0.003601

0.220388	-0.078676	-0.095652	-0.091653	-0.002151	-0.000416	-0.003053
0.368171	0.070237	0.075657	0.054664	-0.002814	-0.001758	-0.001055
0.448710	0.009899	0.022917	0.009691	-0.000288	-0.000577	0.000577

Table S9. Frequency (ω_q), the first-order electronic-vibrational coupling strength of HOMO ($\kappa^{1(n)}_{q-HOMO}$), LUMO ($\kappa^{1(n)}_{q-LUMO}$) and LUMO+1 ($\kappa^{1(n)}_{q-LUMO+1}$) and the second-order coupling strength of HOMO ($\kappa^{2(n)}_{q-HOMO}$), LUMO ($\kappa^{2(n)}_{q-LUMO}$) and LUMO+1 ($\kappa^{2(n)}_{q-LUMO+1}$), for PDI.

PDI						
ω_q (eV)	$\kappa^{1(n)}_{q-HOMO}$ (eV)	$\kappa^{1(n)}_{q-LUMO}$ (eV)	$\kappa^{1(n)}_{q-LUMO+1}$ (eV)	$\kappa^{2(n)}_{q-HOMO}$ (eV)	$\kappa^{2(n)}_{q-LUMO}$ (eV)	$\kappa^{2(n)}_{q-LUMO+1}$ (eV)
0.002828	0.000893	-0.001116	0.000000	0.000000	0.024928	0.016619
0.004688	-0.000317	0.000317	0.000000	-0.004175	0.012524	0.000000
0.011048	-0.000466	0.000815	0.000466	0.000000	0.009046	0.004523
0.011222	0.000000	0.000000	0.000000	-0.005505	-0.005505	0.000000
0.017304	0.000000	0.000000	0.000000	-0.002058	-0.004115	-0.004115
0.017638	0.000000	0.000000	-0.000159	-0.002120	-0.008480	-0.003180
0.018053	-0.000154	-0.000231	-0.000385	0.000990	0.001981	0.000990
0.023313	0.000000	0.000000	0.000000	-0.003210	0.006421	0.000000
0.026580	0.000304	-0.000152	0.000152	0.000000	0.002883	0.000961
0.028819	-0.013589	0.008276	-0.009507	0.000521	0.005212	0.002606
0.042719	0.000055	0.000000	0.000000	-0.002566	-0.003079	-0.001026
0.043295	0.000000	0.000000	0.000000	-0.001281	-0.005123	-0.002561
0.048366	0.000000	0.000000	0.000000	0.000000	0.000977	0.002930
0.049630	-0.018568	-0.024138	-0.011744	0.000000	-0.000359	0.000718
0.055537	-0.000115	0.000058	0.000000	-0.000552	0.004418	0.003313
0.059466	-0.005980	0.002505	-0.002607	-0.001742	0.001742	0.000871
0.068868	0.001168	0.021434	0.024456	0.001353	-0.000541	0.001623
0.081884	0.023890	0.006719	0.030334	0.000000	0.000000	0.000257
0.092713	-0.009335	-0.004969	0.002861	-0.000840	0.001679	0.002519
0.092714	-0.012351	-0.006560	0.003891	-0.001023	-0.002729	-0.001365
0.104179	0.013163	0.010955	-0.008872	-0.000578	-0.002603	-0.002024
0.134864	-0.003898	0.026099	-0.000734	-0.002659	0.000000	0.001064
0.160728	0.000000	0.000076	0.000190	0.002418	0.001934	0.026594
0.162565	-0.002062	0.029749	-0.018354	-0.000886	-0.003987	-0.001772
0.165498	0.053749	-0.032567	0.011221	-0.000999	0.000499	-0.000999
0.175546	0.004985	0.072538	0.025199	-0.000985	-0.000394	-0.000985
0.177602	-0.000103	0.000103	0.000103	0.003983	0.003098	0.015489
0.178482	-0.041617	0.034626	0.007731	-0.000651	-0.000434	-0.000434
0.185503	0.030443	-0.010734	0.016502	0.000000	0.000557	0.000669
0.196038	0.000000	0.000030	0.000000	-0.000603	0.000754	0.013575
0.201751	0.042470	-0.051215	0.037744	0.000345	-0.000345	-0.000517
0.204769	0.004121	-0.075331	-0.052504	0.000358	0.000000	-0.000716
0.208016	-0.000046	0.000000	-0.000068	-0.003384	0.000347	0.019437
0.224228	-0.043543	-0.055538	-0.074292	-0.001148	-0.000725	-0.000423

Table S10. Frequency (ω_q), the first-order electronic-vibrational coupling strength of HOMO ($\kappa^{1(n)}_{q-HOMO}$), LUMO ($\kappa^{1(n)}_{q-LUMO}$) and LUMO+1 ($\kappa^{1(n)}_{q-LUMO+1}$) and the second-order coupling strength of HOMO ($\kappa^{2(n)}_{q-HOMO}$), LUMO ($\kappa^{2(n)}_{q-LUMO}$) and LUMO+1 ($\kappa^{2(n)}_{q-LUMO+1}$), for PDIHs.

PDIHs						
ω_q (eV)	$\kappa^{1(n)}_{q-HOMO}$ (eV)	$\kappa^{1(n)}_{q-LUMO}$ (eV)	$\kappa^{1(n)}_{q-LUMO+1}$ (eV)	$\kappa^{2(n)}_{q-HOMO}$ (eV)	$\kappa^{2(n)}_{q-LUMO}$ (eV)	$\kappa^{2(n)}_{q-LUMO+1}$ (eV)
0.008114	0.000712	0.000712	-0.000178	-0.010551	-0.010551	-0.005275
0.010115	0.000969	0.000646	0.000484	-0.004347	-0.008694	0.004347
0.017362	-0.000515	-0.000172	-0.000601	-0.004915	0.000000	-0.001229
0.020595	0.000000	0.000000	0.000000	0.002727	0.002727	0.002727
0.020643	0.000094	-0.000188	-0.000188	0.001473	0.001473	0.004419
0.022557	-0.000117	-0.000234	0.000117	-0.002279	-0.002279	0.002279
0.025786	0.000655	0.000262	-0.000393	-0.008579	-0.002860	0.005719
0.027309	0.000099	0.000000	-0.000497	-0.001648	0.000000	0.001648
0.028772	-0.004289	0.013443	0.008898	0.000000	0.000000	-0.000683
0.030326	0.000365	-0.000638	-0.000182	-0.002769	-0.001385	-0.002769
0.030901	-0.002870	0.004528	0.009120	0.000000	0.000678	0.000000
0.035500	-0.000122	0.000244	0.000244	0.002484	0.002484	0.002484
0.035728	0.000109	0.000327	0.000109	0.001976	0.003951	0.001976
0.036521	0.000116	-0.000232	-0.000232	0.002237	0.002237	0.002237
0.041502	0.000000	0.000477	0.000204	0.003864	0.003092	-0.000773
0.046174	-0.005336	0.006837	0.005947	0.001545	0.001030	0.000515
0.049451	-0.001504	0.018158	-0.010973	-0.000517	-0.001034	0.001551
0.055038	0.003577	0.002831	0.007153	-0.000823	0.000823	-0.000411
0.059296	-0.000323	-0.003443	-0.001506	0.001447	0.000000	0.002894
0.061026	0.000000	0.000000	0.000000	-0.001618	0.003236	0.001618
0.068059	0.025879	0.010589	0.020500	0.000897	0.000000	0.001495
0.071012	-0.007456	-0.000244	-0.004093	-0.002375	0.000000	-0.001187
0.079297	0.000203	0.000608	0.000270	0.001522	0.004566	0.002283
0.080753	0.003329	0.005145	0.012711	0.000000	-0.000424	0.001272
0.085307	0.001994	0.003869	0.012126	0.000530	0.001061	0.000796
0.091086	0.005275	0.005275	0.004998	-0.001049	0.000000	-0.000787
0.092525	0.000147	-0.000073	0.000073	0.002690	0.002690	0.005380
0.102932	0.005732	0.008228	0.002404	-0.001424	0.000356	-0.001068
0.104751	-0.003556	-0.020731	-0.009108	0.000940	-0.000313	-0.000313
0.130485	0.015371	0.025029	-0.007593	0.000000	0.000453	0.000679
0.135352	-0.008730	0.008314	-0.001403	0.000900	0.000000	0.000450
0.142402	0.024891	0.032278	-0.004503	0.000853	0.000427	0.000427
0.145017	-0.016774	-0.012697	0.009668	0.000565	0.002827	0.001696
0.147693	-0.000134	-0.000134	-0.000604	0.007515	0.002254	0.005260
0.150748	-0.018749	-0.007065	-0.022621	0.000000	-0.000769	0.000000
0.154246	0.002097	0.022911	0.033667	0.001446	-0.000482	0.000964
0.159202	-0.039556	0.007391	0.022586	-0.002913	-0.000583	-0.000583
0.162244	-0.024349	0.052358	-0.026225	0.000788	0.000945	-0.001418
0.166199	0.031895	0.010722	-0.021769	-0.001466	-0.000489	0.000489
0.177119	0.044268	-0.020390	-0.026561	-0.000245	0.000000	-0.000245
0.180273	0.027950	-0.008267	-0.034893	-0.000427	0.000640	-0.000854
0.182415	0.015768	-0.007642	-0.016339	0.000322	0.000322	0.001929
0.187275	-0.003159	0.014666	0.015630	0.000000	0.002396	-0.000553
0.192754	-0.027978	-0.020884	-0.021340	-0.000135	0.001082	-0.000947
0.196038	0.014741	0.023362	0.024440	-0.000806	0.001007	0.002014
0.201516	-0.046902	0.000333	-0.054154	0.000129	0.000515	0.002573
0.204631	0.029814	-0.046164	0.039613	-0.000128	0.002312	-0.002055
0.205437	0.011686	0.010433	0.041815	-0.000618	0.000495	0.000495
0.208949	-0.071405	-0.054812	-0.025086	-0.000229	0.003779	-0.003207
0.222096	-0.054913	-0.059474	-0.078921	-0.001201	-0.000772	-0.001287

Table S11. Frequency (ω_q), the first-order electronic-vibrational coupling strength of HOMO ($\kappa^{1(n)}_{q-HOMO}$), LUMO ($\kappa^{1(n)}_{q-LUMO}$) and LUMO+1 ($\kappa^{1(n)}_{q-LUMO+1}$) and the second-order coupling strength of HOMO ($\kappa^{2(n)}_{q-HOMO}$), LUMO ($\kappa^{2(n)}_{q-LUMO}$) and LUMO+1 ($\kappa^{2(n)}_{q-LUMO+1}$), for TDI.

TDI						
ω_q (eV)	$\kappa^{1(n)}_{q-HOMO}$ (eV)	$\kappa^{1(n)}_{q-LUMO}$ (eV)	$\kappa^{1(n)}_{q-LUMO+1}$ (eV)	$\kappa^{2(n)}_{q-HOMO}$ (eV)	$\kappa^{2(n)}_{q-LUMO}$ (eV)	$\kappa^{2(n)}_{q-LUMO+1}$ (eV)
0.001575	0.000000	0.000000	0.000000	0.000000	0.060767	0.000000
0.003578	0.000000	0.000000	0.000000	0.000000	0.036116	0.000000
0.010135	0.000000	0.000000	0.000000	0.000000	0.009129	0.000000
0.010161	0.000000	0.000000	0.000000	0.000000	0.013257	0.000000
0.010328	0.000000	0.000000	0.000000	0.000000	0.008635	0.000000
0.011391	0.000000	-0.000554	-0.000277	-0.012804	0.000000	-0.003201
0.021254	0.000000	0.000000	0.000000	0.000000	0.006606	0.000000
0.022362	0.012011	-0.007701	0.006601	0.002802	0.004203	0.007005
0.023371	-0.000282	0.000000	-0.000282	0.002952	0.002952	0.001476
0.034347	0.000000	0.000539	0.000308	0.001977	0.000989	0.000989
0.048048	0.012793	0.016994	0.009093	-0.000655	0.000000	-0.000655
0.059636	0.000000	0.000292	0.000073	-0.001778	-0.003557	-0.002668
0.061257	-0.011677	-0.004520	-0.016385	0.000657	0.001971	0.001314
0.062279	-0.006251	-0.010593	-0.018489	0.002165	0.001443	0.001443
0.069505	0.003731	-0.017748	-0.010796	-0.001597	0.000000	-0.000532
0.082409	-0.014194	-0.006817	-0.012014	0.000000	0.000000	0.000000
0.092277	-0.012218	-0.005127	-0.002182	-0.000992	0.000000	-0.000496
0.101489	-0.010139	-0.009614	0.007750	0.001132	0.000566	0.001132
0.115623	0.010177	0.006044	0.005373	0.000445	0.001334	0.001334
0.136827	0.002199	-0.023094	-0.002906	-0.003085	0.000000	-0.004113
0.144114	0.004689	-0.001796	0.009179	0.003318	-0.004977	-0.001659
0.153533	-0.005417	-0.003729	0.009005	0.000000	-0.001650	-0.000825
0.160818	0.010911	-0.032088	0.005240	0.000859	-0.000859	0.000859
0.163713	-0.051044	0.048335	-0.003565	0.003388	-0.000847	0.001694
0.172453	0.014206	0.038942	0.031635	-0.000687	0.000687	0.000343
0.173698	-0.004973	0.045021	-0.001534	0.000933	-0.000466	-0.000466
0.178597	0.029717	-0.018615	0.001147	-0.000457	-0.000685	-0.000457
0.179566	-0.000342	-0.025540	-0.037776	0.000305	0.000610	0.000000
0.185068	0.017153	0.001442	0.014892	0.000000	0.000253	0.000253
0.200291	-0.034384	0.061779	-0.008875	0.000919	0.000919	-0.001225
0.203183	-0.000656	0.043112	0.027572	-0.000496	-0.000248	-0.000248
0.206699	-0.028480	-0.024218	-0.052456	-0.000800	0.000600	0.000600
0.223612	0.036724	0.041702	0.052108	-0.000467	-0.000351	-0.000117

Table S12. Frequency (ω_q), the first-order electronic-vibrational coupling strength of HOMO ($\kappa^{1(n)}_{q-HOMO}$), LUMO ($\kappa^{1(n)}_{q-LUMO}$) and LUMO+1 ($\kappa^{1(n)}_{q-LUMO+1}$) and the second-order coupling strength of HOMO ($\kappa^{2(n)}_{q-HOMO}$), LUMO ($\kappa^{2(n)}_{q-LUMO}$) and LUMO+1 ($\kappa^{2(n)}_{q-LUMO+1}$), for TDIHs.

TDIHs						
ω_q (eV)	$\kappa^{1(n)}_{q-HOMO}$ (eV)	$\kappa^{1(n)}_{q-LUMO}$ (eV)	$\kappa^{1(n)}_{q-LUMO+1}$ (eV)	$\kappa^{2(n)}_{q-HOMO}$ (eV)	$\kappa^{2(n)}_{q-LUMO}$ (eV)	$\kappa^{2(n)}_{q-LUMO+1}$ (eV)
0.002282	0.000000	0.000000	0.000263	0.000000	0.000000	0.011525
0.002633	-0.000572	0.000000	-0.000572	-0.013617	0.000000	0.013617
0.005972	0.000577	0.000577	0.000000	0.000000	0.012314	0.000000
0.008555	0.000000	0.000000	-0.000591	0.000000	0.000000	0.000000
0.010127	-0.000334	0.000000	-0.000334	-0.004654	0.000000	-0.004654
0.011042	0.001161	0.000145	0.000726	0.003512	-0.003512	0.003512
0.011524	-0.000944	-0.000590	-0.001180	0.009290	-0.006967	0.011612
0.020419	0.000000	0.000000	-0.000310	0.000000	0.003549	0.003549
0.020520	0.000000	0.000209	0.000000	0.000000	0.001822	0.000000
0.021473	-0.000245	0.000000	0.000123	-0.002501	0.005003	-0.002501
0.022468	0.004001	-0.014724	-0.006802	-0.001067	0.001067	-0.004269
0.023446	0.002633	-0.000905	-0.004855	0.005644	-0.003386	0.004515
0.025969	0.000000	0.000000	0.000136	0.000000	0.000000	0.003079
0.029305	0.000000	0.000000	0.000000	0.000000	0.003082	0.000000
0.032925	0.000000	0.000000	0.000000	0.000000	0.003805	0.000000
0.035207	0.000000	0.000000	0.000000	0.000000	0.005349	0.000000
0.036365	0.000000	0.000000	0.000000	0.000000	0.006686	0.000000
0.038086	0.000000	0.000000	0.000000	0.000000	0.002581	0.000000
0.039153	0.000000	0.000000	0.000332	0.000000	0.004085	0.000000
0.042206	0.000368	-0.005293	-0.006028	0.001801	0.002702	0.004503
0.044932	-0.002708	0.004393	0.000842	-0.003621	-0.001207	-0.005432
0.048168	0.000840	-0.012283	0.009180	0.000697	0.002090	0.002090
0.049125	-0.000057	0.000000	0.000171	-0.000541	-0.001081	0.002703
0.056837	-0.004130	-0.002344	-0.001507	-0.002076	-0.001038	0.000000
0.061076	-0.002054	-0.007773	-0.004386	0.001028	0.000514	0.000514
0.061757	-0.006980	-0.002448	-0.004428	0.000904	0.000452	0.001357
0.068495	-0.022044	-0.004590	-0.014537	0.000000	0.000000	0.000000
0.069760	-0.006042	-0.000113	-0.002823	0.000000	0.001594	0.000531
0.076046	-0.002816	-0.002608	-0.006728	0.000000	0.000000	-0.000907
0.087862	0.002486	0.003701	0.006407	0.002034	0.000000	0.000000
0.090517	0.003824	0.008214	0.005854	-0.000371	0.001114	0.001857
0.100875	-0.002845	-0.017842	-0.003665	-0.001163	0.000000	-0.001938
0.107336	-0.006243	-0.007840	-0.004888	-0.000390	0.000390	0.001171
0.115569	-0.001539	-0.008883	-0.001759	-0.000322	0.000967	0.001289
0.127022	0.009246	0.013316	-0.004702	-0.000520	-0.000260	-0.000260
0.137178	0.011981	0.010484	0.002052	-0.001026	0.000513	-0.000513
0.138521	0.011351	0.021890	0.003675	-0.000974	0.001948	0.000000
0.144856	-0.018272	-0.016937	0.011526	-0.002469	0.000000	-0.007408
0.147515	0.020478	0.014144	-0.001548	0.002476	0.000000	0.002476
0.148522	0.011718	0.000154	0.003315	0.000991	-0.000991	0.005943
0.151191	0.007963	0.000239	0.022217	0.002114	0.000000	0.001057
0.155530	0.008671	-0.023392	-0.026417	-0.002118	-0.001271	-0.001271
0.159104	-0.046015	0.032248	-0.008604	0.000000	0.000000	-0.000933
0.161430	-0.011535	0.045145	-0.026275	0.000634	0.000000	-0.000634
0.164142	0.024346	-0.006167	-0.012205	-0.000688	-0.000688	-0.000688
0.165860	0.029984	0.002743	-0.009697	0.001357	-0.001357	0.003392
0.170480	-0.000780	0.008667	0.020177	0.001051	0.000000	0.000000
0.172532	-0.013697	0.002144	-0.000569	-0.001915	0.000319	-0.000638
0.174070	-0.006639	0.011803	0.005164	0.000000	0.000453	-0.000453
0.177953	-0.038115	0.004812	0.034696	0.000000	0.000891	-0.000297
0.181652	0.018008	-0.001995	-0.021350	0.001938	-0.001453	0.000000
0.187962	-0.003939	0.010005	0.016386	-0.000259	0.000000	0.000259
0.190769	-0.019698	-0.007647	-0.044858	0.000000	0.001360	-0.000194
0.195752	0.006121	0.008866	0.026933	-0.001376	0.000229	0.000459
0.201391	0.042441	0.009992	0.038952	-0.001006	0.000000	-0.000335
0.203529	-0.042187	0.047499	-0.066218	0.000000	0.000326	0.000488
0.205284	0.012593	-0.008076	-0.016033	0.000000	0.001044	0.000149
0.206551	0.047141	0.016804	0.037559	-0.000579	0.002173	-0.001883
0.210270	0.033548	0.033519	0.036923	-0.000282	0.000988	0.000847
0.222084	0.042640	0.044134	0.061363	-0.000535	-0.000963	-0.000321

In our calculation, the couplings, $u_1(u_2)$ between orbitals are expressed by the ratio of corresponding transition dipole moments, and the time-dependent normalization coefficients at t = 0, $A_i(0)$, $B_i(0)$ and $C_i(0)$ are expressed by the ratio of the square of the oscillator strengths. In order to maintain the consistency of calculation conditions, the transition dipole moment and oscillator strength of each system are calculated at the level of TD-HSE06/6-311G**, as follows:

Table S13. Excitation energies (E_{Sn}), transition electric dipole moments (μ_{Sn}), oscillator strengths (f_n) and main transition contribution (Ctr_n) of singlet(doublet) ground state (S_0 , D_0) to the first singlet (doublet) state (S_1 , D_1) and S_0 (D_0) to S_2 (D_2), for NDI, NDIH-diradical, $NDIH_3O$-diradical, $NDINH_4$-diradical by TDDFT.

	$E_{S1\ (D1)}\ (eV)$	$\mu_{S1\ (D1)}\ (atomic.u.)$	f_1	Ctr_1	$E_{S2\ (D2)}\ (eV)$	$\mu_{S2\ (D2)}\ (atomic.u.)$	f_2	Ctr_2
NDI	3.472	1.788	0.272	H -> L 98.0 %	-	-	-	-
NDIH-radical	2.097	0.741	0.028	H -> L 84.3%	3.062	1.559	0.182	H -> L+1 58.8%
NDIH-diradical	2.400	1.668	0.164	H -> L 99.9 %	3.721	1.937	0.342	H -> L+1 93.0%
$NDIH_3O$-diradical	2.413	1.733	0.178	H -> L 99.7%	3.685	1.971	0.351	H -> L+1 93.5%
$NDINH_4$-diradical	2.408	1.791	0.189	H -> L 99.7%	3.640	1.925	0.330	H -> L+1 93.3%
PDI	2.466	3.204	0.620	H -> L 100.2%	-	-	-	-
PDIHs	2.182	2.477	0.328	H -> L 96.2%	2.846	2.493	0.4335	H -> L+1 89.9%
TDI	1.972	4.696	1.0654	H -> L 101.9%	-	-	-	-
TDIHs	2.006	4.057	0.8086	H -> L 97.1%	2.492	2.520	0.3876	H -> L+1 91.5%

Then, the orbital energy couplings, u_1 (u_2) and the time-dependent normalization coefficients at t = 0, $A_i(0)$, $B_i(0)$ and $C_i(0)$ can be expressed below:

Table S14. The orbital couplings of HOMO to LUMO/SOMO (u_1) and HOMO to LUMO+1/LUMO (u_2). $A_i(0)$, $B_i(0)$, $C_i(0)$ represent the time-dependent normalization coefficients at t = 0 of HOMO, LUMO/SOMO and LUMO+1/LUMO. respectively.

	u_1 (eV)	u_2 (eV)	$A_i(0)$	$B_i(0)$	$C_i(0)$
NDI	0.100	0.000	0.000	1.000	0.000
NDIH-radical	0.100	0.210	0.000	0.153	0.989
NDIH-diradical	0.100	0.116	0.000	0.569	0.823
$NDIH_3O$-diradical	0.100	0.114	0.000	0.452	0.892
$NDINH_4$-diradical	0.100	0.107	0.000	0.500	0.868
PDI	0.100	0.000	0.000	1.000	0.000
PDIHs	0.100	0.101	0.000	0.603	0.797
TDI	0.100	0.000	0.000	1.000	0.000
TDIHs	0.100	0.062	0.000	0.902	0.432

For NDIHs anion vertical dimer, it is found that at the NIR region, the main transition contribution is S_0-S_5/S_6, which mainly comes from the orbital transition of HOMO to LUMO+5/LUMO+6. In the meantime, the energies of LUMO+5 and LUMO+6 are basically equal. Therefore, only two orbitals, HOMO and LUMO+5, are considered in the Davydov ansatz calculation. In this case, many second-order coupling strengths are 0, and a few large second-order coupling strengths make the calculation time much longer. For improving the calculation efficiency, only the first-order electronic-vibrational coupling strength which satisfies $\left|\kappa_q^{1(n)}/\omega_q\right|>0.05$ is considered here.

Table S15. Orbital energies (eV) of HOMO and LUMO+5 of NDIHs anion vertical dimer. The orbital couplings of HOMO to LUMO+5 (u_5(eV)). Ai (0), Bi (0) represent the time-dependent normalization coefficients at t = 0 of HOMO and LUMO+5. respectively.

	HOMO	LUMO+5	u_5	$A_i(0)$	$B_i(0)$
NDIHs anion vertical dimer	8.312	9.491	0.100	0.000	1.000

Table S16. Excitation energies (E_{Sn}), transition electric dipole moments (μ_{Sn}), oscillator strengths (f_n) and main transition contribution (Ctr_n) of singlet ground state (S_0) to the fifth singlet excited state (S_5) and S_0 to S_6 , for NDIHs anion vertical dimer by TDDFT.

	$E_{S5}\ (eV)$	$\mu_{S5}\ (atomic.u.)$	f_5	Ctr_5	$E_{S6}\ (eV)$	$\mu_{S6}\ (atomic.u.)$	f_6	Ctr_6
NDIHs anion vertical dimer	1.097	4.039	0.109	H -> L+5 51.3 %	1.097	4.037	0.109	H -> L+6 51.3 %

Table S17. Frequency (ω_q), the first-order electronic-vibrational coupling strength of HOMO ($\kappa^{1(n)}_{q-HOMO}$), LUMO+5 ($\kappa^{1(n)}_{q-LUMO+5}$)for NDIHs anion vertical dimer.

NDIHs anion vertical dimer		
ω_q (eV)	$\kappa^{1(n)}_{q-HOMO}$ (eV)	$\kappa^{1(n)}_{q-LUMO+5}$ (eV)
0.005945594	-0.000545	-0.000545
0.005980865	-0.000542	-0.000407
0.007829937	0.009523	-0.016552
0.011185968	-0.000618	0.000865
0.01334476	-0.006071	-0.000559
0.014427371	-0.014691	0.004407
0.01492939	0.001939	-0.002644
0.021166998	0.000728	0.003277
0.021700213	-0.001482	0.012819
0.022205531	0.001321	-0.006395
0.031839991	-0.002185	0.005244
0.033908544	0.004277	0.007999
0.036424009	0.000181	-0.001875
0.040420313	-0.012289	0.011899
0.040782971	0.002793	0.007373
0.042912815	-0.007725	0.007159
0.046121137	0.003536	0.006138
0.047545633	-0.001805	-0.010935
0.050102697	0.000513	-0.006211
0.050742398	-0.003456	-0.005209
0.057365225	-0.013155	-0.014714
0.063903777	0.008247	-0.011415
0.070665156	0.009153	-0.016932
0.072766988	-0.008085	0.015353
0.082230044	-0.004161	0.007555
0.087319555	0.005286	-0.01088
0.090715983	-0.005138	0.003613
0.091780761	0.014101	-0.012418
0.096705001	-0.014009	0.013345
0.115551324	0.008381	0.004437
0.14845263	-0.005571	0.01372
0.174277906	0.017975	-0.008586
0.18233743	-0.009426	-0.000943
0.182546453	0.012563	-0.012375
0.188288265	-0.025259	0.024244
0.192007536	-0.022459	0.030014
0.204423973	0.00669	-0.016886

Experimental Spectrum Characterization

The UV-vis absorption spectrum characterization was performed on NDI ionized derivatives with D_2O, because OH overtone bands when using H_2O would interfere the absorption spectrum characterization and D_2O could eliminate the absorption peak of H_2O.[31] The NDI ionized derivatives solution in hydrazine hydrate was heated in glovebox in 80°C to remove solvent, afterward the NDI ionized derivatives powder was dissolved in degassed D_2O in concentration of 10^{-4} mol L^{-1} in quartz cuvette, which was sealed to avoid contact with O_2 and characterized in ambient condition.

Supporting Figures and Tables

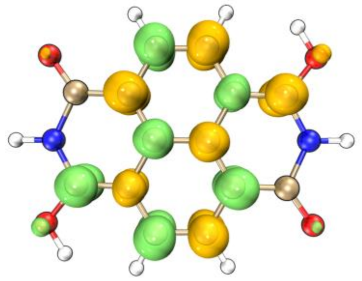

Fig. S2. spin density diagram of NDIH-diradical spin polarized singlet state at the UHF/6-311G** level. Red, blue, gold and white spheres represent O, N, C and H, respectively. The isovalue is 0.015 atomic.u.. Green and yellow regions denote positive and negative spin density parts, respectively.

Although the diradical characteristics of NDIH-diradical cannot be described at the DFT, HSE06/6-311G** level (Table S18), in the UHF level, the symmetry breaking wavefunction can be constructed and diradical characteristics can be showed. At the UHF/6-311G** level,[32] the energy of spin polarized singlet state of NDIH-diradical is -943.4424 atomic.u., lower than -943.4220 atomic.u. of singlet state. The $<S^2>$ is 1.4261. The diradical characteristic value, y based on the occupation numbers of UHF natural orbital (UNO) is 0.9731, which is very close to the ideal diradical characteristic value y = 1.[33]

Table S18. Energies and spin square operator expectations of NDI, NDIH-diradical, $NDIH_3O$-diradical and $NDINH_4$-diradical. E_{S0} and E_{T0} represent the ground state energies of singlet and triplet states, respectively. $<S_{S0}^2>$ and $<S_{T0}^2>$ represent the spin square operator expectations of singlet and triplet states, respectively.

	E_{S0} (atomic.u.)	$<S_{S0}^2>$	E_{T0} (atomic.u.)	$<S_{T0}^2>$
NDI	-946.873	0.000	-946.787	2.028
NDIH-diradical	-948.030	0.000	-947.982	2.024
$NDIH_3O$-diradical	-1100.811	0.000	-1100.761	2.023
$NDINH_4$-diradical	-1061.099	0.000	-1061.049	2.022

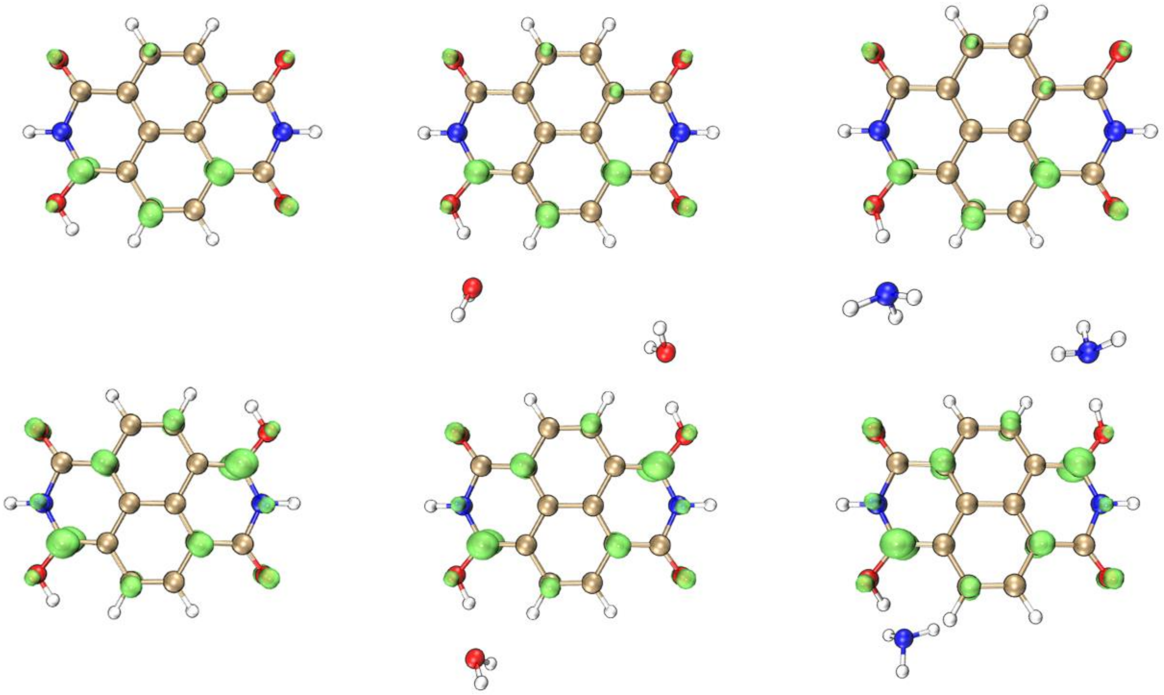

Fig. S3. spin density diagram of NDIH-radical doublet state, NDIH-diradical triplet state, $NDIH_3O$-radical doublet state, $NDIH_3O$-diradical triplet state, $NDINH_4$-radical doublet state and $NDINH_4$-diradical triplet state. All isovalues are 0.015 atomic.u.. Green regions denote positive spin density parts.

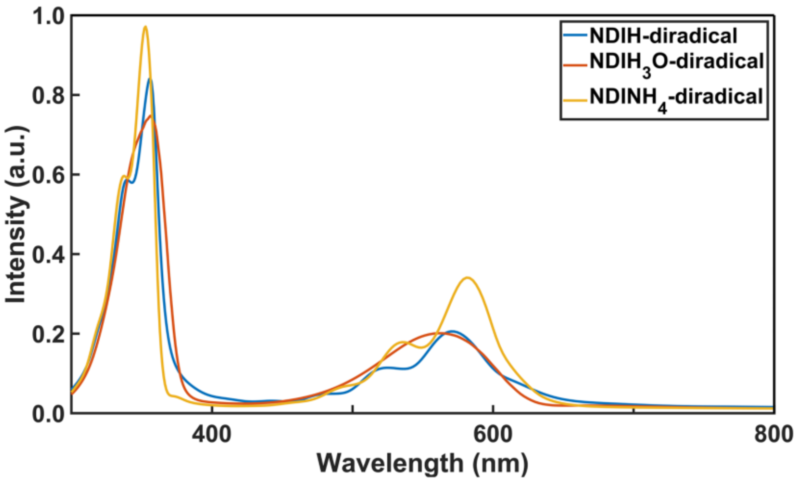

Fig. S4. UV-Vis absorption spectra of NDIH-diradical, $NDIH_3O$-diradical and $NDINH_4$-diradical obtained by Davydov ansatz.

Table S19. Energies and spin square operator expectations of PDI, PDIHs, TDI and TDIHs. E_{S0} and E_{T0} represent the ground state energies of singlet and triplet states, respectively. $<S_{S0}^2>$ and $<S_{T0}^2>$ represent the spin square operator expectations of singlet and triplet states, respectively.

	E_{S0} (atomic.u.)	$<S_{S0}^2>$	E_{T0} (atomic.u.)	$<S_{T0}^2>$
PDI	-1330.060	0.000	-1330.007	2.039
PDIHs	-1331.221	0.000	-1331.174	2.029
TDI	-1713.246	0.000	-1713.208	2.048
TDIHs	-1714.408	0.000	-1714.366	2.035

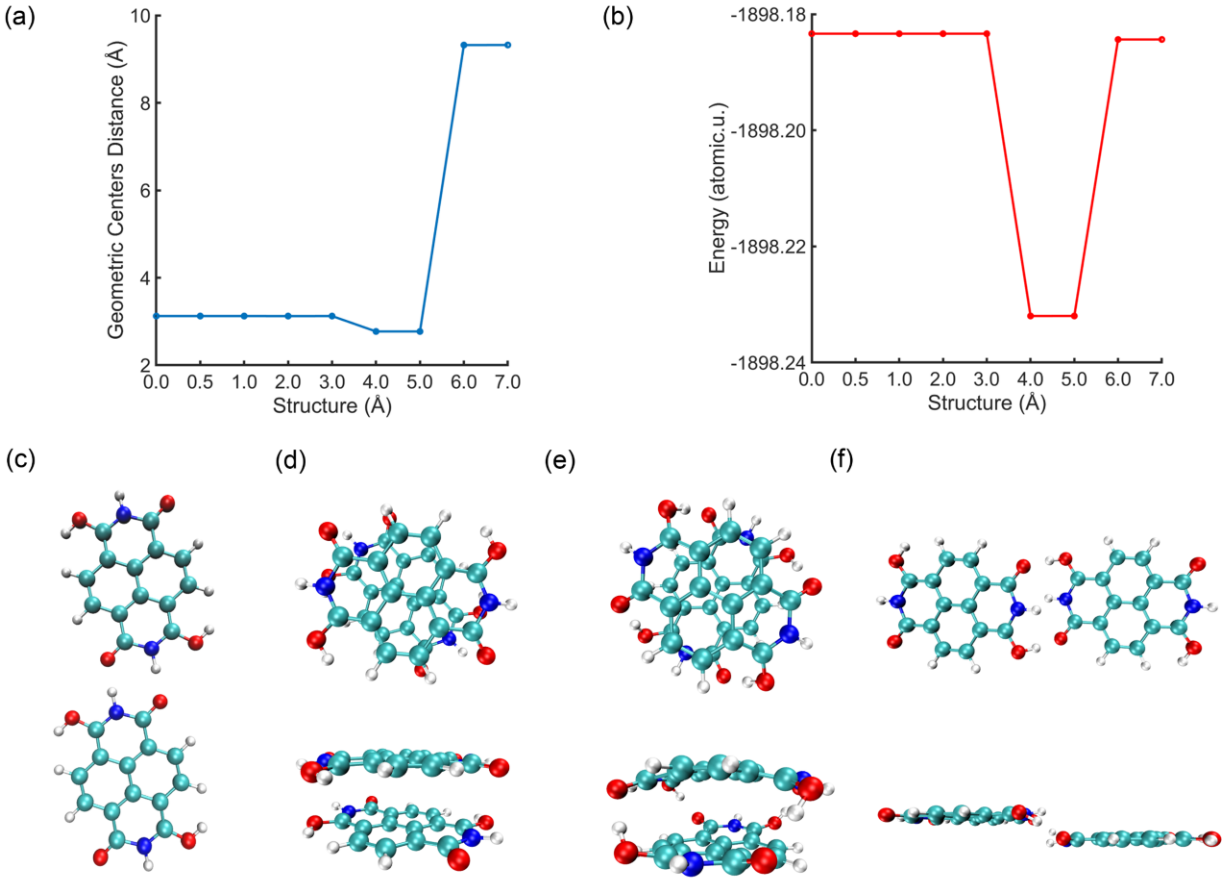

Fig. S5. (a) Geometric center distance changes of nine NDIHs vertical dimers. (b) energy changes of nine NDIHs vertical dimers. (c) Optimized structure of NDIHs horizontal dimer. (d) Optimized structure of NDIHs dimer by initial vertical stacking. (e) Optimized structure of NDIHs dimer by an initial slip of 4.0 Å of the second layer in the Y direction. (f) Optimized structure of NDIHs dimer by an initial slip of 6.0 Å of the second layer in the Y direction.

For obtaining NDIHs vertical dimer, we set the vertical stacking distance to 3.45 Å according to the common π-π stacking distance,[34] and then set the sliding distances along the Y direction as 0.0 to 7.0 Å to move the monomer of the second layer, respectively. After geometric optimization, these structures can be divided by geometric center distance and energy into three groups (Fig. S5). The second group which involves the structures with an initial slip of 4.0 and 5.0 Å in the Y direction, has the lowest energy and the shortest geometric center distance. When the slip distance exceeds a limit of 6.0 Å, the attraction between the two monomers decreases, and the dimer dissociates, resulting in a sharp increase in the geometric center distance and energy. The geometric center distance of the first group structures is between the second group and the third group, but the energy is the highest. We can find that the structures of the first group and the second group are relatively similar. The difference is that the bending of the second group structures is more intense. It is considered that the strong radical-radical bonding reduces the energy of the second group. Therefore, radical-radical bonding is discussed in detail in our article.

In the following context, we only consider the vertical dimer with the lowest energy, taking the NDIHs dimer with initial slip of 4.0 Å as an example, and call it NDIHs vertical dimer.

For further explore NDIHs dimers, we compare the ESP distributions and orbital images of NDIHs horizontal dimer and NDIHs vertical dimer. It is observed that ESP distribution range of NDIHs vertical dimer is narrower, but the percentage of positive ESP in the total area is larger than that in the horizontal case (Fig. S6). For NDIHs horizontal dimer, the energy of two adjacent energy levels is similar. For example, the energy of HOMO-1 is -4.806 eV, the energy of HOMO is -4.780 eV, the energy of LUMO is -2.160 eV, and the energy of LUMO+1 is -2.133 eV. This makes the electron density distribution of adjacent orbitals very similar (Fig. S7a). The difference is that the wavefunction phase of one of two monomers in adjacent orbitals is opposite. For NDIHs vertical dimer, the isosurface of charge density is also similar for different phases between two adjacent orbitals, such as HOMO-1 and HOMO. The overlaps of electron clouds in the orbitals of LUMO, LUMO+1, LUMO+2 and LUMO+3 further prove the formation of pancake bonding.

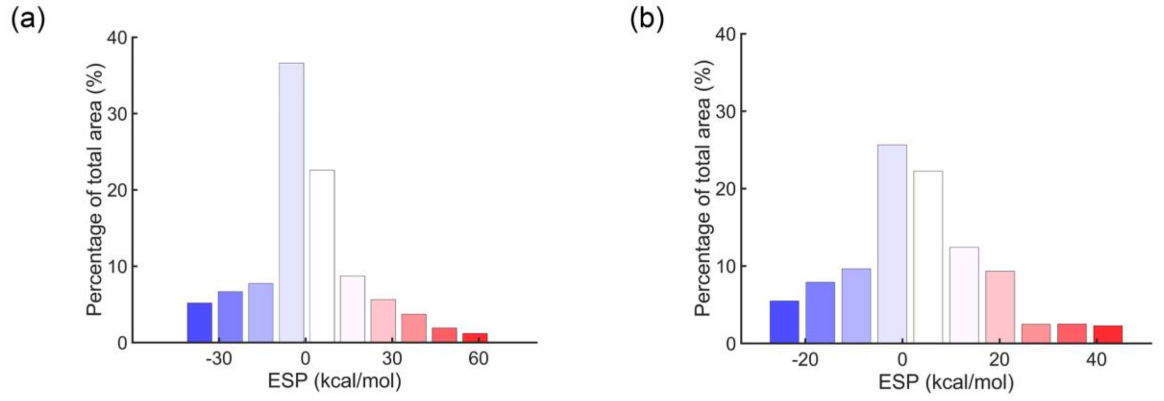

Fig. S6. (a) ESP distribution of NDIHs horizontal dimer. (b) ESP distribution of NDIHs vertical dimer.

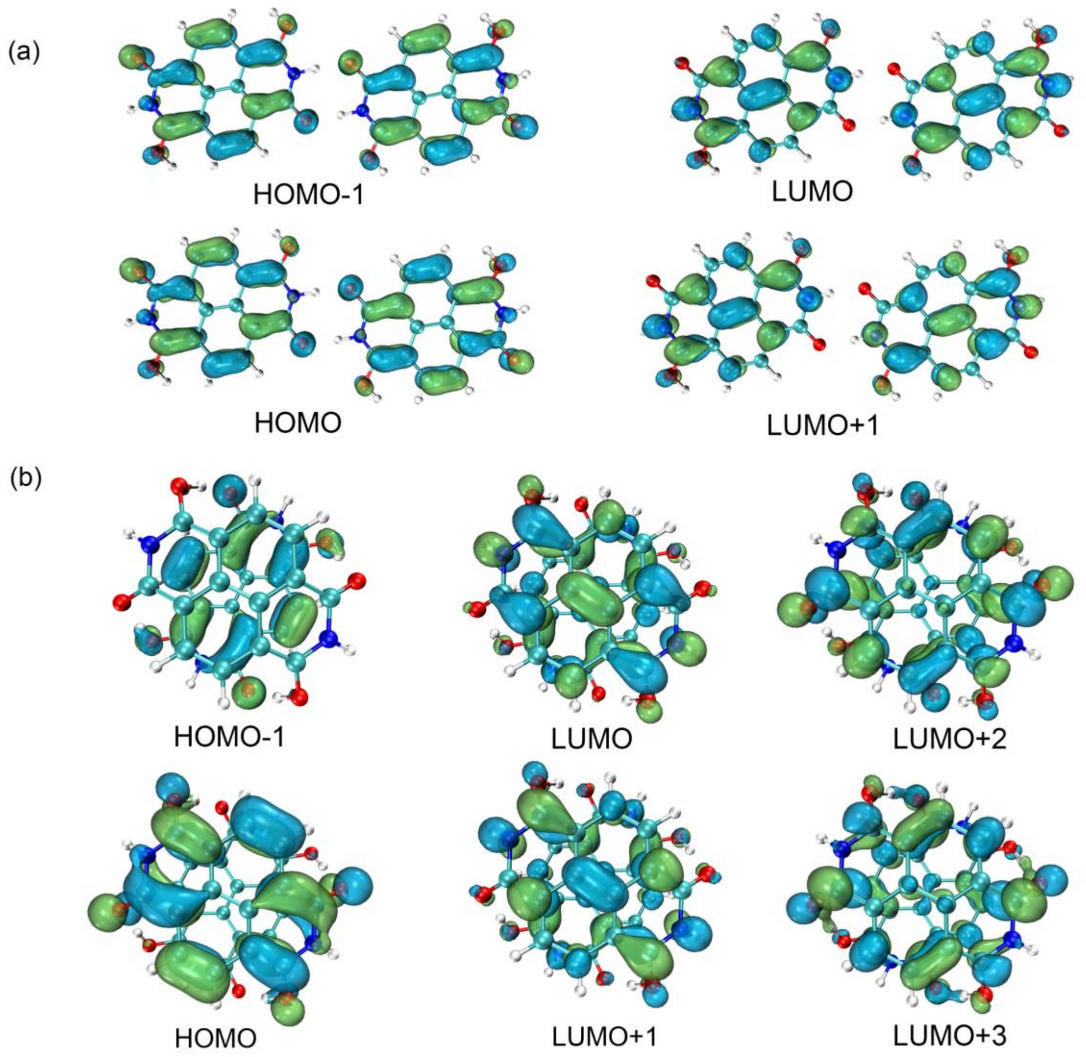

Fig. S7. (a) Molecular orbital images of NDI horizontal dimer (isovalue = 0.03 atomic.u.). (b) Molecular orbital images of NDI vertical dimer (isovalue = 0.03 atomic.u.).

Table S20. Interaction energies of NDIHs horizontal dimer and NDIHs vertical dimer, obtained by B3LYP-D3BJ/ 6-311+G** and sSAPT0/jun-cc-pVDZ, respectively.

	B3LYP-D3BJ/ 6-311+G** (kcal/mol)	sSAPT0/jun-cc-pVDZ (kcal/mol)
NDIHs horizontal dimer	-22.73	-24.84
NDIHs vertical dimer	-73.70	-83.03

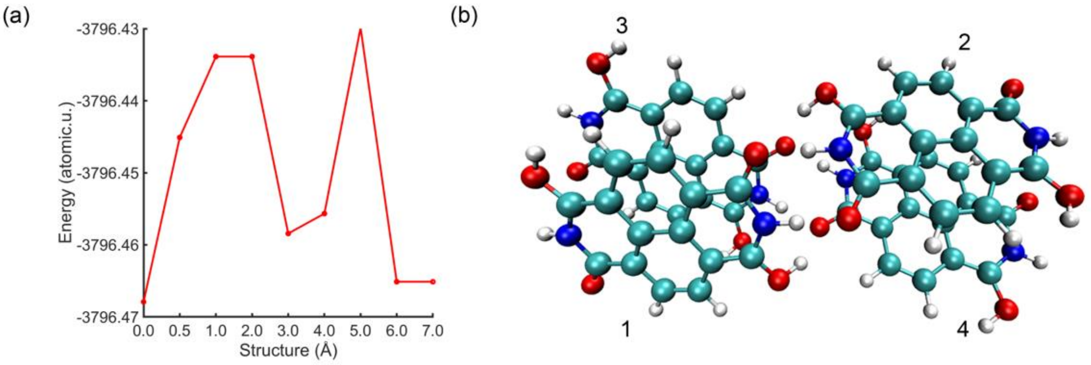

Fig. S8. (a) Energy changes of nine NDIHs tetramers. (b) Optimized structure of NDIHs tetramer by initial vertical stacking. 1,2,3,4 represents the monomer setting in SAPT calculation, respectively.

For NDIHs tetramer, we also set the initial vertical stacking distance to 3.45 Å and the sliding distances between two layers along the Y direction as 0 to 7.0 Å. The energy of 0.0 Å, which represents the initial vertical stacking, is the lowest (Fig. S8).

The molecular orbital from HOMO-3 to LUMO+3 can be simply divided into two groups (Fig. S9). The first group is HOMO-3 to HOMO. The orbitals in this group are similar to the superposition of monomer NDIHs LUMO orbits. The electronic clouds of HOMO-3 and HOMO-2 are concentrated on the two NDIHs at the bottom, and those of HOMO-1 and HOMO are concentrated at the top. The electron density of HOMO-3 and HOMO-1 overlaps in opposite phases, while that of HOMO-2 and HOMO overlap in the same phase. The second group is from LUMO to LUMO+3, and their distribution is similar to that of the first group with different electron density. The phase of LUMO and LUMO+2 is same, while that of LUMO+1 and LUMO+3 is opposite. ESP distribution of NDIHs tetramer shows the negative region is larger (Fig. S10).

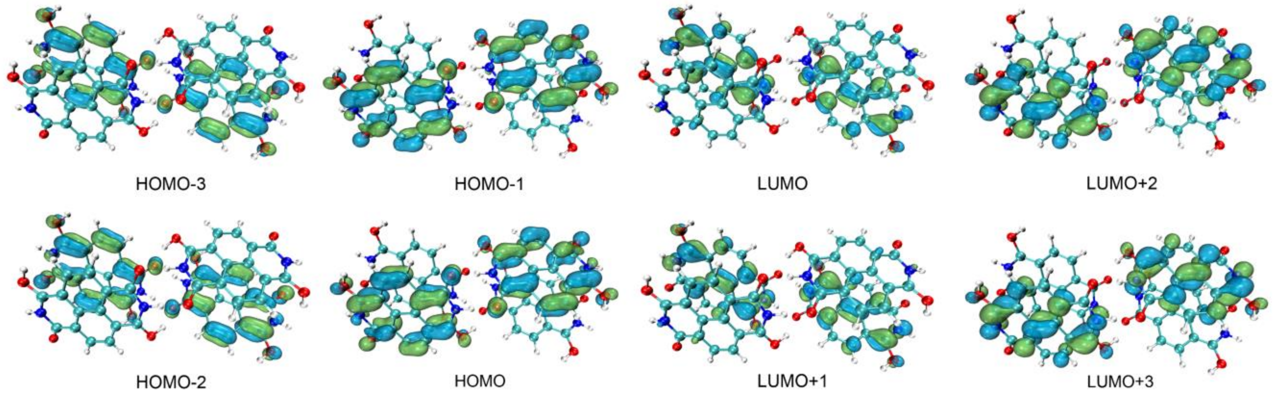

Fig. S9. Molecular orbital images of NDIHs tetramer by initial vertical stacking (isovalue = 0.03 atomic.u.).

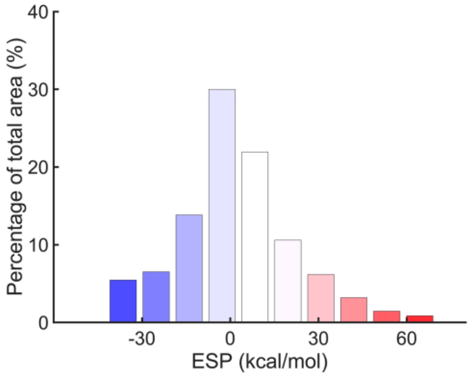

Fig. S10. ESP distribution of NDIHs tetramer by initial vertical stacking.

Table S21. Interaction energy of NDIHs tetramer obtained by B3LYP-D3BJ/ 6-311+G** and sSAPT0/jun-cc-pVDZ, respectively.

	B3LYP-D3BJ/ 6-311+G** (kcal/mol)	sSAPT0/jun-cc-pVDZ (kcal/mol)
Tetramer	-118.58	-
Pair 1-2[a]	-26.86	-29.24
Pair 1-3	-18.42	-21.38
Pair 1-4	-17.16	-19.20
Pair 2-3	-17.16	-19.20
Pair 2-4	-18.42	-21.38
Pair 3-4	-21.96	-24.16

[a] The pair division can be seen in Fig. S8.

For NDI, PDI and TDI, ESP minimum values decrease with the increase of atomic numbers. Starting from the first strip whose central coordinate is positive, the percentage of positive ESP of NDI is 68.15%, that of PDI is 72.42%, and that of TDI is 75.27% (Fig. 1e; Fig. S11).

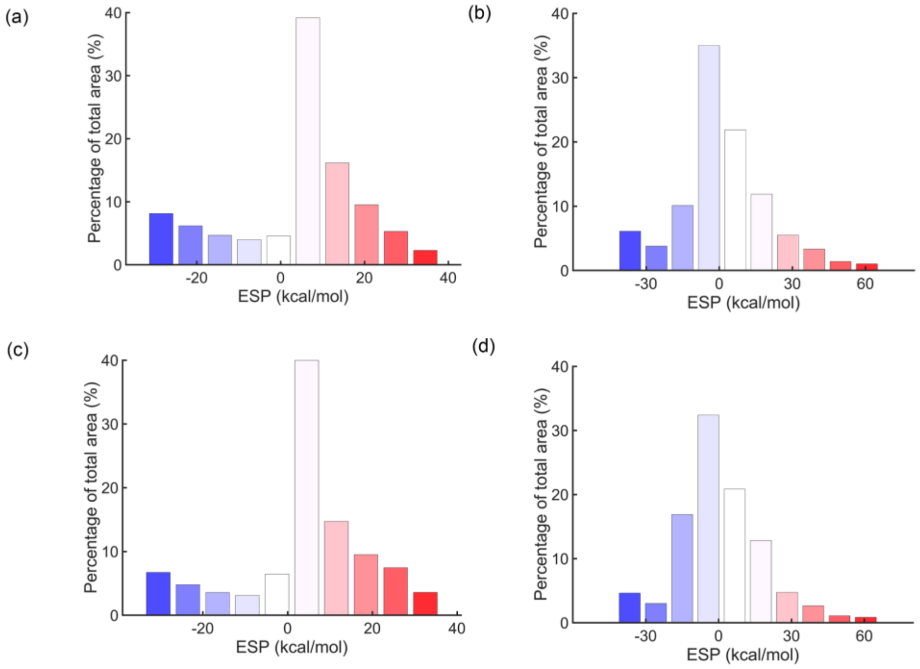

Fig. S11. (a) ESP distribution of PDI. (b) ESP distribution of PDIHs. (c) ESP distribution of TDI. (d) ESP distribution of TDIHs.

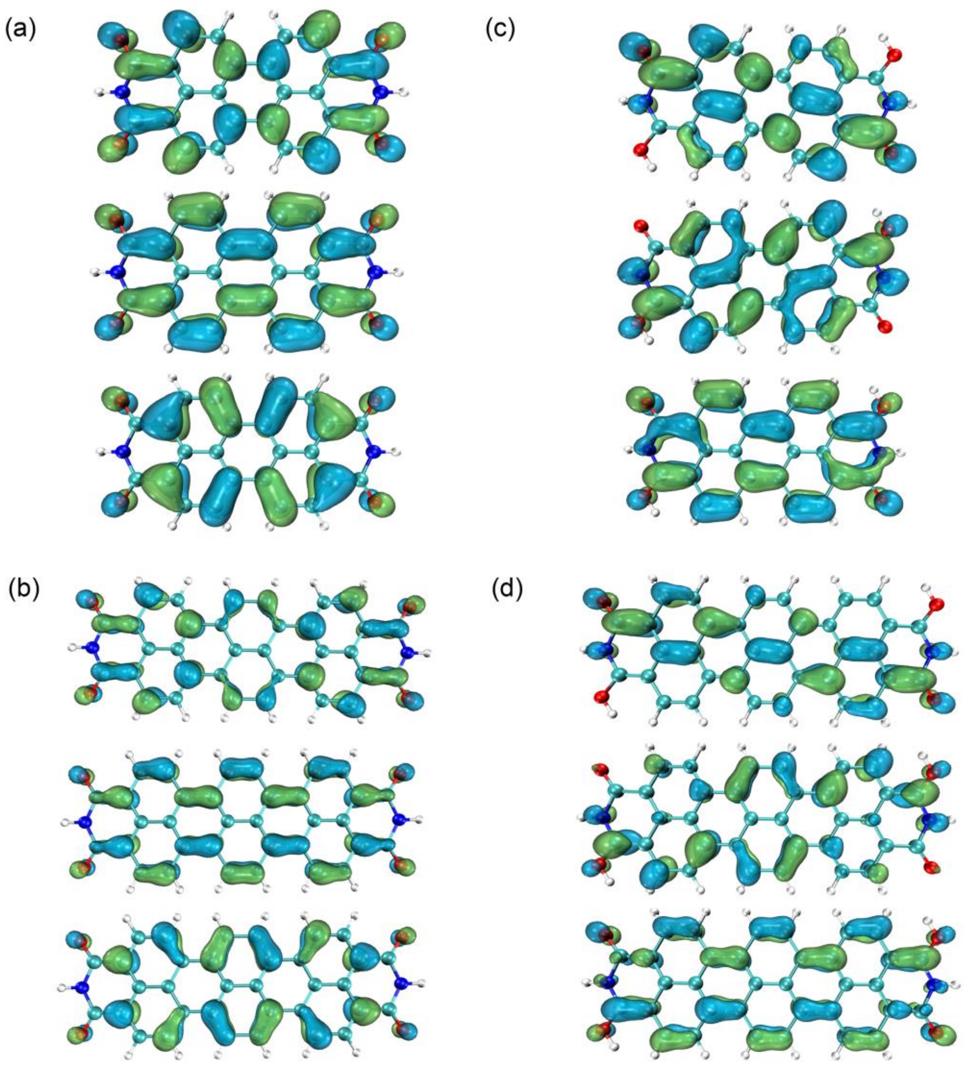

Fig. S12. (a) Molecular orbital images of PDI (isovalue = 0.03 atomic.u.). (b) Molecular orbital images of PDIHs (isovalue = 0.03 atomic.u.). (c) Molecular orbital images of TDI (isovalue = 0.035 atomic.u.). (d) Molecular orbital images of TDIHs (isovalue = 0.035 atomic.u.).

Table S22. Optimized Cartesian coordinates (Å) of NDIH-radical at HSE06/6-311G** level in the gas phase, charge = 0, spin multiplicity = 1.

NDIH-radical			
atom	x	y	z
C	1.40342600	-1.23912500	0.00000800
C	0.67981500	-2.43497700	0.00000400
C	-0.69575700	-2.42810300	0.00000700
C	-1.41065800	-1.20206300	0.00003100
C	-0.69027100	0.03732400	0.00005000
C	0.73027600	0.00805200	0.00002600
C	1.43916400	1.22596200	0.00001400
C	0.76406800	2.44281000	0.00001500
C	-0.61937000	2.47363000	0.00003600
C	-1.34811000	1.28001700	0.00006700
C	2.86807400	-1.28453500	-0.00002600
C	2.91446700	1.21700400	0.00000600
C	-2.80066300	-1.11801100	-0.00001600
C	-2.80908100	1.33733000	0.00016900
O	3.52194500	-2.30864200	-0.00002300
O	3.59862000	2.21842600	-0.00005100
O	-3.49082400	2.33950600	-0.00011200
O	-3.64686800	-2.14972100	-0.00010100
N	-3.42505200	0.07771600	0.00002600
N	3.49196200	-0.04305800	-0.00000600
H	1.23899700	-3.36340300	-0.00002000
H	-1.21894700	-3.38063300	-0.00002000
H	1.35232200	3.35312800	0.00001100
H	-1.16787400	3.40800900	0.00005300
H	-4.43712400	0.08872300	-0.00006100
H	4.50381200	-0.06127100	-0.00004300
H	-3.15482800	-2.97560500	-0.00012200

Table S23. Optimized Cartesian coordinates (Å) of NDIH-diradical at HSE06/6-311G** level in the gas phase, charge = 0, spin multiplicity = 1.

NDIH-diradical			
atom	x	y	z
C	-1.34728600	1.28203900	0.00002000
C	-0.58595700	2.48723600	0.00000800
C	0.76791200	2.44896300	-0.00000200
C	1.45850600	1.19431700	0.00000800
C	0.71624400	-0.03397300	0.00002600
C	-0.71624600	0.03397500	0.00002800
C	-1.45850300	-1.19431600	0.00003200
C	-0.76791100	-2.44896000	0.00007500
C	0.58596000	-2.48723400	0.00006700
C	1.34728300	-1.28203500	0.00003000
C	-2.78957400	1.37780700	0.00002000
C	-2.83753900	-1.08028400	-0.00002700
C	2.83753700	1.08028400	-0.00001300
C	2.78957600	-1.37780300	0.00001100
O	-3.46078300	2.39492500	0.00002500
O	-3.70996300	-2.09592000	-0.00007200
O	3.46077400	-2.39492900	-0.00006500
O	3.70996800	2.09591600	-0.00004700
N	3.43090100	-0.12671000	0.00000300
N	-3.43090000	0.12670500	-0.00003600
H	-1.13306000	3.42207300	0.00000100
H	1.31930000	3.38599100	-0.00001900
H	-1.31929700	-3.38599000	0.00016900
H	1.13306400	-3.42207000	0.00010300
H	4.44233300	-0.16168800	-0.00005000
H	-4.44233200	0.16169200	-0.00006600
H	-3.23016600	-2.92857500	-0.00033000
H	3.23017600	2.92857400	0.00000600

Table S24. Optimized Cartesian coordinates (Å) of $NDIH_3O$-diradical at HSE06/6-311G** level in the gas phase, charge = 0, spin multiplicity = 1.

$NDIH_3O$-diradical			
atom	x	y	z
C	1.75100200	-0.62728700	0.00000000
C	2.48361400	0.59453700	0.00000000
C	1.84235200	1.78763800	0.00000000
C	0.41160000	1.84350200	0.00000000
C	-0.35150100	0.62642700	0.00000000
C	0.35144000	-0.62640400	0.00000000
C	-0.41164200	-1.84353200	0.00000000
C	-1.84241700	-1.78765700	0.00000000
C	-2.48366000	-0.59456000	0.00000000
C	-1.75107600	0.62730200	0.00000000
C	2.48361400	-1.87073500	0.00000000
C	0.30778500	-3.03149100	0.00000000
C	-0.30778200	3.03154800	0.00000000
C	-2.48366000	1.87072200	0.00000000
O	3.69706300	-2.01210000	0.00000000
O	-0.17016400	-4.27107000	0.00000000
O	-3.69708700	2.01223000	0.00000000
O	0.17028300	4.27092200	0.00000000
O	-2.77518500	-5.02491400	0.00000000
O	2.77527600	5.02482300	0.00000000
N	-1.65710800	3.00241000	0.00000000
N	1.65713700	-3.00238400	0.00000000
H	3.56477400	0.52335300	0.00000000
H	2.41304500	2.70842100	0.00000000
H	-2.41306700	-2.70845000	0.00000000
H	-3.56482400	-0.52338100	0.00000000
H	-2.14225000	3.89019600	0.00000000
H	2.14220000	-3.89021000	0.00000000
H	-1.14499700	-4.33278200	0.00000000
H	-3.34586400	-4.93086100	0.76590600
H	-3.34586400	-4.93086100	-0.76590600
H	1.14511600	4.33256000	0.00000000
H	3.34601500	4.93131900	0.76592300
H	3.34601500	4.93131900	-0.76592300

Table S25. Optimized Cartesian coordinates (Å) of $NDINH_4$-diradical at HSE06/6-311G** level in the gas phase, charge = 0, spin multiplicity = 1.

$NDINH_4$-diradical			
atom	x	y	z
C	-0.51140000	1.78381800	-0.14619200
C	0.72650200	2.39891000	-0.49508200
C	1.86023100	1.66225300	-0.59906200
C	1.83758500	0.25419400	-0.33729900
C	0.59415500	-0.39956100	-0.04903300
C	-0.59415200	0.39956000	0.04903200
C	-1.83758200	-0.25419500	0.33729900
C	-1.86022900	-1.66225400	0.59906100
C	-0.72649900	-2.39891100	0.49508100
C	0.51140300	-1.78381900	0.14619100
C	-1.69065300	2.60996200	-0.03571900
C	-2.97649300	0.54132100	0.37190100
C	2.97649600	-0.54132200	-0.37190000
C	1.69065600	-2.60996300	0.03572000
O	-1.75341500	3.82440300	-0.15667000
O	-4.21920400	0.12225600	0.56393100
O	1.75341800	-3.82440400	0.15667100
O	4.21920700	-0.12225700	-0.56392700
N	2.86093000	-1.87830000	-0.21212100
N	-2.86092700	1.87829900	0.21212300
N	-4.74918600	-2.09610300	-0.85876600
N	4.74916600	2.09611200	0.85876200
H	0.71187000	3.46342400	-0.69763200
H	2.78625300	2.12139300	-0.92464400
H	-2.78625000	-2.12139400	0.92464300
H	-0.71186700	-3.46342600	0.69763100
H	3.71280500	-2.42302300	-0.24256200
H	-3.71280200	2.42302200	0.24256500
H	-4.38592600	-0.76188100	0.11141700
H	-5.19942100	-2.86808200	-0.37909700
H	-3.88483500	-2.45115400	-1.25631700
H	-5.35253600	-1.82658900	-1.62830900
H	4.38592100	0.76188800	-0.11142900
H	3.88481300	2.45115700	1.25631500
H	5.19940300	2.86809900	0.37910700
H	5.35251200	1.82658700	1.62830400

Table S26. Optimized Cartesian coordinates (Å) of NDIHs horizontal dimer at HSE06/6-311G** level in the gas phase, charge = 0, spin multiplicity = 1.

NDIHs horizontal dimer			
atom	x	y	z
C	-0.73141000	3.37274600	0.00000000
C	-2.08940600	3.82583900	0.00000000
C	-2.37786200	5.15005400	0.00000000
C	-1.32536100	6.12997800	0.00000000
C	0.04993200	5.70222800	0.00000000
C	0.32972600	4.29369500	0.00000000
C	1.69691000	3.86372400	0.00000000
C	2.75099500	4.83485900	0.00000000
C	2.46570900	6.16287200	0.00000000
C	1.11270800	6.61612700	0.00000000
C	-0.45745300	1.96251000	0.00000000
C	1.91419400	2.48682300	0.00000000
C	-1.54783500	7.49537100	0.00000000
C	0.85823800	8.04496800	0.00000000
O	-1.32529200	1.07072800	0.00000000
O	3.12133000	1.89591300	0.00000000
O	1.68804000	8.94178100	0.00000000
O	-2.75099500	8.10397800	0.00000000
N	-0.51583100	8.36882100	0.00000000
N	0.89244600	1.61523900	0.00000000
H	-2.86604500	3.07321200	0.00000000
H	-3.41821100	5.46107700	0.00000000
H	3.79068100	4.52231800	0.00000000
H	3.24408900	6.91396800	0.00000000
H	-0.72706600	9.35928100	0.00000000
H	1.10431100	0.58858100	0.00000000
H	3.80893400	2.56987100	0.00000000
H	-3.44781400	7.43906800	0.00000000
C	-1.11270800	-6.61612700	0.00000000
C	-2.46570900	-6.16287200	0.00000000
C	-2.75099500	-4.83485900	0.00000000
C	-1.69691000	-3.86372400	0.00000000
C	-0.32972600	-4.29369500	0.00000000
C	-0.04993200	-5.70222800	0.00000000
C	1.32536100	-6.12997800	0.00000000
C	2.37786200	-5.15005400	0.00000000
C	2.08940600	-3.82583900	0.00000000
C	0.73141000	-3.37274600	0.00000000
C	-0.85823800	-8.04496800	0.00000000
C	1.54783500	-7.49537100	0.00000000
C	-1.91419400	-2.48682300	0.00000000
C	0.45745300	-1.96251000	0.00000000
O	-1.68804000	-8.94178100	0.00000000
O	2.75099500	-8.10397800	0.00000000
O	1.32529200	-1.07072800	0.00000000
O	-3.12133000	-1.89591300	0.00000000
N	-0.89244600	-1.61523900	0.00000000
N	0.51583100	-8.36882100	0.00000000
H	-3.24408900	-6.91396800	0.00000000
H	-3.79068100	-4.52231800	0.00000000
H	3.41821100	-5.46107700	0.00000000
H	2.86604500	-3.07321200	0.00000000
H	-1.10431100	-0.58858100	0.00000000
H	0.72706600	-9.35928100	0.00000000
H	3.44781400	-7.43906800	0.00000000
H	-3.80893400	-2.56987100	0.00000000

Table S27. Optimized Cartesian coordinates (Å) of NDIHs vertical dimer of initial slip 0.0 Å at HSE06/6-311G** level in the gas phase, charge = 0, spin multiplicity = 1.

NDIHs vertical dimer of initial slip 0.0 Å			
atom	x	y	z
C	1.70686300	-0.75456700	1.63751300
C	1.39700000	-2.14847400	1.63474800
C	0.10781400	-2.56846600	1.63063400
C	-0.96782300	-1.61993600	1.63053100
C	-0.68617600	-0.21120700	1.66181900
C	0.68656100	0.20919700	1.66195000
C	0.96821100	1.61795400	1.63175300
C	-0.10742900	2.56648100	1.63269300
C	-1.39661300	2.14648800	1.63655500
C	-1.70648500	0.75257600	1.63819500
C	3.09518000	-0.35384200	1.56791700
C	2.30101600	1.97918500	1.54157100
C	-2.30063700	-1.98111400	1.54021300
C	-3.09480200	0.35189500	1.56838700
O	4.07642000	-1.08317100	1.49597300
O	2.77189300	3.23695000	1.41630400
O	-4.07605700	1.08127100	1.49709500
O	-2.77150800	-3.23879400	1.41413600
N	-3.27580700	-1.04802500	1.53949900
N	3.27618800	1.04609500	1.54009600
H	2.22088500	-2.84761000	1.60196400
H	-0.09469300	-3.63426500	1.61569800
H	0.09506900	3.63229300	1.61859800
H	-2.22050000	2.84565100	1.60438100
H	-4.23732200	-1.35308500	1.45214900
H	4.23769500	1.35121500	1.45286400
H	2.02933200	3.84660400	1.33824900
H	-2.02895500	-3.84839400	1.33561000
C	0.82955500	-1.66586600	-1.63648100
C	-0.30067600	-2.53945900	-1.66303700
C	-1.56216300	-2.04032300	-1.66995300
C	-1.78409100	-0.62209500	-1.64076100
C	-0.66258200	0.27899000	-1.66319500
C	0.66218700	-0.27697400	-1.66353000
C	1.78370400	0.62409700	-1.64043700
C	1.56177100	2.04236000	-1.66843000
C	0.30028000	2.54147400	-1.66087900
C	-0.82994200	1.66785500	-1.63490000
C	2.15095500	-2.24840900	-1.51873000
C	3.03944600	0.04565300	-1.55271000
C	-3.03983200	-0.04371000	-1.55247700
C	-2.15131000	2.25029800	-1.51641600
O	2.42092000	-3.43649300	-1.41831900
O	4.20625100	0.71817400	-1.48245800
O	-2.42125200	3.43828900	-1.41482200
O	-4.20665300	-0.71625700	-1.48283600
N	-3.18956000	1.29414900	-1.49289700
N	3.18920000	-1.29226900	-1.49436900
H	-0.10512500	-3.60327300	-1.66448900
H	-2.40139100	-2.72932300	-1.67797900
H	2.40101000	2.73134900	-1.67613500
H	0.10472100	3.60528800	-1.66144000
H	-4.11356700	1.66144200	-1.29595300
H	4.11321300	-1.65973400	-1.29777600
H	4.03609100	1.65978500	-1.59301400
H	-4.03661700	-1.65771400	-1.59485700

Table S28. Optimized Cartesian coordinates (Å) of NDIHs vertical dimer of initial slip 4.0 Å at HSE06/6-311G** level in the gas phase, charge = 0, spin multiplicity = 1.

NDIHs vertical dimer of initial slip 4.0 Å			
atom	x	y	z
C	0.43877200	-1.81058000	-1.65511000
C	1.86578200	-1.74801700	-1.73964600
C	2.50770000	-0.55286000	-1.73721900
C	1.75987400	0.66601600	-1.64181500
C	0.33068300	0.63672000	-1.72720700
C	-0.33158600	-0.63772500	-1.72727100
C	-1.76086700	-0.66695800	-1.64193700
C	-2.50859800	0.55193200	-1.73745900
C	-1.86664200	1.74707200	-1.73984100
C	-0.43962300	1.80966600	-1.65519500
C	-0.19938600	-3.05192300	-1.32169400
C	-2.35793100	-1.88930300	-1.35858700
C	2.35727700	1.88869300	-1.35950700
C	0.19843600	3.05121700	-1.32277300
O	0.37697100	-4.10167800	-0.96817400
O	-3.64444000	-2.07533200	-1.06267400
O	-0.37762200	4.10132400	-0.97031500
O	3.64361100	2.07458900	-1.06338800
N	1.58879000	3.00750500	-1.29712200
N	-1.58963000	-3.00826100	-1.29577800
H	2.41210800	-2.68119900	-1.77563000
H	3.58757000	-0.49990700	-1.77462900
H	-3.58848900	0.49902800	-1.77489300
H	-2.41308700	2.68017400	-1.77607500
H	2.04522700	3.85511500	-0.98134000
H	-2.04564300	-3.85507100	-0.97723200
H	-3.93085800	-1.42133800	-0.36266900
H	3.92928200	1.42165100	-0.36164800
C	1.81075900	0.43951800	1.65546700
C	1.74810600	1.86665100	1.73880000
C	0.55299600	2.50856900	1.73646900
C	-0.66608700	1.76081700	1.64231500
C	-0.63668700	0.33156200	1.72771700
C	0.63776500	-0.33074000	1.72762400
C	0.66702800	-1.75995400	1.64229900
C	-0.55189900	-2.50774300	1.73717400
C	-1.74704100	-1.86580400	1.73984100
C	-1.80957600	-0.43876500	1.65576700
C	3.05205700	-0.19857900	1.32217100
C	1.88943600	-2.35718700	1.35883500
C	-1.88858100	2.35789800	1.35939200
C	-3.05131000	0.19923300	1.32301100
O	4.10171500	0.37772600	0.96822100
O	2.07525300	-3.64341100	1.06231800
O	-4.10091300	-0.37709100	0.96998300
O	-2.07516800	3.64422100	1.06310300
N	-3.00758800	1.58952700	1.29706700
N	3.00850700	-1.58892900	1.29657700
H	2.68132300	2.41295500	1.77400200
H	0.50014100	3.58849500	1.77310800
H	-0.49904000	-3.58765400	1.77437600
H	-2.68015400	-2.41225200	1.77577300
H	-3.85443600	2.04547400	0.97852000
H	3.85507700	-2.04477800	0.97716000
H	1.42162200	-3.92927800	0.36129200
H	-1.42158000	3.93033800	0.36235800

Table S29. Optimized Cartesian coordinates (Å) of NDIHs vertical dimer of initial slip 6.0 Å at HSE06/6-311G** level in the gas phase, charge = 0, spin multiplicity = 1.

NDIHs vertical dimer of initial slip 6.0 Å			
atom	x	y	z
C	6.15024500	1.22597000	0.02742800
C	5.50610800	2.49678200	-0.00879000
C	4.15830900	2.58727100	-0.16340900
C	3.37730800	1.39910300	-0.30968800
C	3.99294200	0.10800800	-0.26664200
C	5.41481900	0.03818100	-0.08817700
C	6.04262400	-1.25926500	-0.02865200
C	5.23543500	-2.44662000	-0.11619600
C	3.89194800	-2.36421800	-0.26210300
C	3.24269300	-1.08819400	-0.34929300
C	7.59484000	1.18355500	0.19737400
C	7.41523200	-1.27688400	0.12765800
C	1.99407200	1.44175900	-0.48611900
C	1.83094200	-1.02498100	-0.50984000
O	8.34821700	2.13710100	0.30852100
O	8.19547500	-2.37400400	0.20331100
O	1.02938700	-1.98799000	-0.53868500
O	1.30235700	2.56692900	-0.51125300
N	1.31998500	0.27235600	-0.64710400
N	8.11927100	-0.12537900	0.22661100
H	6.12891400	3.37517500	0.09500900
H	3.65343000	3.54321000	-0.18930700
H	5.69581400	-3.42813500	-0.05743100
H	3.27246400	-3.24834100	-0.32591500
H	0.34292400	0.32997900	-0.90154700
H	9.12387300	-0.18726500	0.33900900
H	7.65581500	-3.16372100	0.09138300
H	0.37511000	2.41006200	-0.16509900
C	-3.24246600	1.08771500	0.35059000
C	-3.89134200	2.36395600	0.26367000
C	-5.23472200	2.44679400	0.11705200
C	-6.04219600	1.25970100	0.02849000
C	-5.41476700	-0.03794500	0.08776200
C	-3.99301600	-0.10823700	0.26698700
C	-3.37777000	-1.39952400	0.30980500
C	-4.15899900	-2.58741500	0.16257500
C	-5.50669200	-2.49648600	0.00723500
C	-6.15044700	-1.22548100	-0.02879300
C	-1.83085400	1.02404000	0.51206700
C	-1.99462600	-1.44265600	0.48693700
C	-7.41469800	1.27775900	-0.12859500
C	-7.59494800	-1.18258900	-0.19954100
O	-1.02903600	1.98680300	0.54204300
O	-1.30327400	-2.56804500	0.51182300
O	-8.34850000	-2.13590100	-0.31152700
O	-8.19462300	2.37511100	-0.20418700
N	-8.11900200	0.12647700	-0.22847700
N	-1.32030200	-0.27352600	0.64887000
H	-3.27166400	3.24788800	0.32827200
H	-5.69480200	3.42846500	0.05853500
H	-3.65440000	-3.54350300	0.18836000
H	-6.12969300	-3.37465300	-0.09730500
H	-9.12352300	0.18871000	-0.34140200
H	-0.34339200	-0.33148700	0.90386500
H	-0.37560900	-2.41115700	0.16685700
H	-7.65481600	3.16464700	-0.09169800

Table S30. Optimized Cartesian coordinates (Å) of NDIHs tetramer of initial slip 0.0 Å at HSE06/6-311G** level in the gas phase, charge = 0, spin multiplicity = 1.

NDIHs tetramer of initial slip 0.0 Å			
atom	x	y	z
C	-3.38873400	-0.24768000	1.90939700
C	-4.25040100	0.81575600	2.32330500
C	-5.59452400	0.70752600	2.18841700
C	-6.17930700	-0.46341000	1.59760200
C	-5.33483100	-1.54431800	1.16456100
C	-3.91757900	-1.43059800	1.36446200
C	-3.07085800	-2.49260000	0.91331300
C	-3.63777600	-3.63891300	0.26715800
C	-4.98383500	-3.71473200	0.06577900
C	-5.84959700	-2.66787100	0.50602400
C	-1.96859000	-0.08197500	1.99844400
C	-1.69511600	-2.31197900	1.05403300
C	-7.53215700	-0.59184300	1.33884900
C	-7.26280000	-2.74827800	0.18399500
O	-1.39040000	0.96392700	2.35598500
O	-0.81993100	-3.21089100	0.60145400
O	-7.81587100	-3.62566900	-0.46260400
O	-8.48021400	0.30807700	1.67128400
N	-8.01807300	-1.66333100	0.67796100
N	-1.21114100	-1.18612800	1.61758700
H	-3.78765800	1.70406200	2.72914800
H	-6.22169100	1.53913700	2.49441900
H	-2.97405200	-4.43965200	-0.03315500
H	-5.44087200	-4.56574800	-0.42243300
H	-8.99702300	-1.65505700	0.41517900
H	-0.18630300	-1.13034500	1.83370800
H	-0.06549200	-2.72987700	0.17599000
H	-8.08765700	0.98703800	2.23019100
C	5.84847100	2.66803500	0.50598400
C	4.98282300	3.71497100	0.06570300
C	3.63678200	3.63940400	0.26734300
C	3.06977900	2.49327000	0.91371600
C	3.91636400	1.43121200	1.36492900
C	5.33360300	1.54470100	1.16481500
C	6.17795700	0.46370000	1.59783300
C	5.59307600	-0.70693000	2.18919400
C	4.24896200	-0.81497400	2.32426100
C	3.38737900	0.24844900	1.91013400
C	7.26166200	2.74819200	0.18381300
C	7.53077400	0.59184500	1.33885800
C	1.69401900	2.31287000	1.05456000
C	1.96723700	0.08289900	1.99922400
O	7.81480800	3.62551500	-0.46281700
O	8.47874800	-0.30822800	1.67115600
O	1.38882300	-0.96292700	2.35662000
O	0.81889900	3.21181200	0.60189900
N	1.20996800	1.18719000	1.61835700
N	8.01681900	1.66321100	0.67783700
H	5.43991000	4.56584900	-0.42270200
H	2.97311300	4.44013200	-0.03311400
H	6.22020000	-1.53841700	2.49562600
H	3.78619200	-1.70300700	2.73067000
H	0.18509800	1.13134100	1.83415100
H	8.99577600	1.65481700	0.41508200
H	8.08609700	-0.98734600	2.22980100
H	0.06508500	2.73073000	0.17526000
C	-3.05291600	1.56936000	-0.91409800
C	-3.20121900	2.86497800	-0.32025800
C	-4.42983000	3.40485700	-0.13411900
C	-5.61510100	2.70115400	-0.53866300
C	-5.49737200	1.40051300	-1.14600000

C	-4.19253100	0.83786900	-1.31405100
C	-4.08532900	-0.47824400	-1.86406500
C	-5.24995100	-1.17673800	-2.30640900
C	-6.47994700	-0.62245300	-2.14354100
C	-6.62099100	0.65787000	-1.53712600
C	-1.76267400	0.98810200	-1.06650800
C	-2.81088300	-1.03878100	-1.89440500
C	-6.89633200	3.17771300	-0.34218500
C	-7.95953000	1.16970000	-1.30284400
O	-0.65579800	1.54061900	-0.81417200
O	-2.52606900	-2.29227300	-2.27672300
O	-9.01403100	0.60232000	-1.54619300
O	-7.23095500	4.35288800	0.23155400
N	-7.97642200	2.44721700	-0.70775200
N	-1.73814900	-0.32352700	-1.51583200
H	-2.31185100	3.38756300	0.00157300
H	-4.50520000	4.37524400	0.34661600
H	-5.15179800	-2.15411900	-2.75997300
H	-7.37878000	-1.14222400	-2.44562700
H	-8.89735600	2.83151100	-0.53541000
H	-0.83117000	-0.82221300	-1.41877100
H	-3.22466200	-2.88649800	-1.95800200
H	-6.43699900	4.87764600	0.38079000
C	6.62231700	-0.65901100	-1.53675500
C	6.48155800	0.62126400	-2.14337300
C	5.25172100	1.17593500	-2.30619500
C	4.08693800	0.47794900	-1.86347200
C	4.19388000	-0.83800400	-1.31301000
C	5.49853000	-1.40116600	-1.14520200
C	5.61591100	-2.70182700	-0.53779600
C	4.43047500	-3.40496000	-0.13274600
C	3.20208000	-2.86436300	-0.31823100
C	3.05412800	-1.56885300	-0.91237100
C	7.96072600	-1.17127900	-1.30273800
C	6.89702700	-3.17884600	-0.34162200
C	2.81262400	1.03880800	-1.89394000
C	1.76398500	-0.98739200	-1.06502500
O	9.01537500	-0.60429400	-1.54628600
O	7.23135000	-4.35415000	0.23204300
O	0.65706300	-1.53960600	-0.81246600
O	2.52816100	2.29218200	-2.27704300
N	1.73971100	0.32408800	-1.51489500
N	7.97728200	-2.44876100	-0.70745900
H	7.38050200	1.14068100	-2.44573500
H	5.15382700	2.15321700	-2.76002900
H	4.50554000	-4.37536100	0.34801100
H	2.31259600	-3.38626800	0.00439100
H	0.83267700	0.82292300	-1.41829500
H	8.89812400	-2.83337700	-0.53534500
H	6.43715300	-4.87829400	0.38214000
H	3.22659200	2.88648400	-1.95808200

References

1 M. J. Frisch, G. W. Trucks, H. B. Schlegel, G. E. Scuseria, M. A. Robb, J. R. Cheeseman, G. Scalmani, V. Barone, G. A. Petersson, H. Nakatsuji, X. Li, M. Caricato, A. v Marenich, J. Bloino, B. G. Janesko, R. Gomperts, B. Mennucci, H. P. Hratchian, J. v Ortiz, A. F. Izmaylov, J. L. Sonnenberg, D. Williams-Young, F. Ding, F. Lipparini, F. Egidi, J. Goings, B. Peng, A. Petrone, T. Henderson, D. Ranasinghe, V. G. Zakrzewski, J. Gao, N. Rega, G. Zheng, W. Liang, M. Hada, M. Ehara, K. Toyota, R. Fukuda, J. Hasegawa, M. Ishida, T. Nakajima, Y. Honda, O. Kitao, H. Nakai, T. Vreven, K. Throssell, J. A. Montgomery Jr., J. E. Peralta, F. Ogliaro, M. J. Bearpark, J. J. Heyd, E. N. Brothers, K. N. Kudin, V. N. Staroverov, T. A. Keith, R. Kobayashi, J. Normand, K. Raghavachari, A. P. Rendell, J. C. Burant, S. S. Iyengar, J. Tomasi, M. Cossi, J. M. Millam, M. Klene, C. Adamo, R. Cammi, J. W. Ochterski, R. L. Martin, K. Morokuma, O. Farkas, J. B. Foresman and D. J. Fox, 2016.

2 X. Xie, Y. Liu, Y. Yao, U. Schollwöck, C. Liu and H. Ma, *The Journal of Chemical Physics*, 2019, **151**, 224101.

3 N. Zhou, Z. Huang, J. Zhu, V. Chernyak and Y. Zhao, *The Journal of Chemical Physics*, 2015, **143**, 014113.

4 L. Chen, M. F. Gelin and W. Domcke, *The Journal of Chemical Physics*, 2019, **150**, 024101.

5 T. Lu and F. Chen, *Journal of Computational Chemistry*, 2012, **33**, 580–592.

6 J. Zhang and T. Lu, *Physical Chemistry Chemical Physics*, 2021, **23**, 20323–20328.

7 T. Lu and F. Chen, *Journal of Molecular Graphics and Modelling*, 2012, **38**, 314–323.

8 S. Manzetti and T. Lu, *Journal of Physical Organic Chemistry*, 2013, **26**, 473–483.

9 T. Lu and S. Manzetti, *Structural Chemistry*, 2014, **25**, 1521–1533.

10 T. Lu and Q. Chen, *Chemistry–Methods*, 2021, **1**, 231–239.

11 W. Humphrey, A. Dalke and K. Schulten, *Journal of Molecular Graphics*, 1996, **14**, 33–38.

12 A. v Krukau, O. A. Vydrov, A. F. Izmaylov and G. E. Scuseria, *The Journal of Chemical Physics*, 2006, **125**, 224106.

13 A. D. Becke, *The Journal of Chemical Physics*, 1993, **98**, 5648–5652.

14 P. J. Stephens, F. J. Devlin, C. F. Chabalowski and M. J. Frisch, *The Journal of Physical Chemistry*, 1994, **98**, 11623–11627.

15 J. M. Crowley, J. Tahir-Kheli and W. A. Goddard, *The Journal of Physical Chemistry Letters*, 2016, **7**, 1198–1203.

16 H. Xiao, J. Tahir-Kheli and W. A. Goddard, *The Journal of Physical Chemistry Letters*, 2011, **2**, 212–217.

17 S. Grimme, S. Ehrlich and L. Goerigk, *Journal of Computational Chemistry*, 2011, **32**, 1456–1465.

18 J. Witte, M. Goldey, B. Neaton and M. Head-gordon, , DOI:10.1021/ct501050s.

19 C. Adamo and D. Jacquemin, *Chemical Society Reviews*, 2013, **42**, 845–856.

20 T. Yanai, D. P. Tew and N. C. Handy, *Chemical Physics Letters*, 2004, **393**, 51–57.

21 T. Clark, J. Chandrasekhar, G. W. Spitznagel and P. V. R. Schleyer, *Journal of Computational Chemistry*, 1983, **4**, 294–301.

22 J. Zeng, S.-B. Qiu, Y.-J. Zhao, X.-B. Yang and Y. Yao, *The Journal of Physical Chemistry Letters*, 2020, **11**, 1194–1198.

23 L. Chen, M. F. Gelin and W. Domcke, *The Journal of Chemical Physics*, 2019, **150**, 024101.

24 N. Zhou, Z. Huang, J. Zhu, V. Chernyak and Y. Zhao, *The Journal of Chemical Physics*, 2015, **143**, 014113.

25 K.-W. Sun and Y. Yao, *The Journal of Chemical Physics*, 2017, **147**, 224905.

26 L. Wang, L. Chen, N. Zhou and Y. Zhao, *The Journal of Chemical Physics*, 2016, **144**, 024101.

27 N. Zhou, Z. Huang, J. Zhu, V. Chernyak and Y. Zhao, *The Journal of Chemical Physics*, 2015, **143**, 014113.

28 J. Zeng, S.-B. Qiu, Y.-J. Zhao, X.-B. Yang and Y. Yao, *The Journal of Physical Chemistry Letters*, 2020, **11**, 1194–1198.

29 L. Chen, M. F. Gelin and W. Domcke, *The Journal of Chemical Physics*, 2019, **150**, 024101.

30 G. A. Worth, H. -D. Meyer and L. S. Cederbaum, *The Journal of Chemical Physics*, 1996, **105**, 4412–4426.

31 J. F. Penneau, B. J. Stallman, P. H. Kasai and L. L. Miller, *Chemistry of Materials*, 1991, **3**, 791–796.

32 J. A. Pople and R. K. Nesbet, *The Journal of Chemical Physics*, 1954, **22**, 571–572.

33 K. Yamaguchi, *Chemical Physics Letters*, 1975, **33**, 330–335.

34 S. Grimme, *Angewandte Chemie International Edition*, 2008, **47**, 3430–3434.
